# Laminar CBV and BOLD response characteristics over time and space in the human primary somatosensory cortex at 7T

**DOI:** 10.1162/IMAG.a.1157

**Published:** 2026-03-27

**Authors:** Sebastian Dresbach, Omer Faruk Gulban, Till Steinbach, Judith Eck, Sriranga Kashyap, Amanda Kaas, Nikolaus Weiskopf, Rainer Goebel, Renzo Huber

**Affiliations:** Faculty of Psychology and Neuroscience, Maastricht University, Maastricht, The Netherlands; Department of Neurophysics, Max Planck Institute for Human Cognitive and Brain Sciences, Leipzig, Germany; Brain Innovation, Maastricht, The Netherlands; Krembil Brain Institute, University Health Network, Toronto, ON, Canada; Felix Bloch Institute for Solid State Physics, Faculty of Physics and Earth System Sciences, Leipzig University, Leipzig, Germany; Wellcome Centre for Human Neuroimaging, Institute of Neurology, University College London, London, United Kingdom; National Institutes of Health, Bethesda, MD, United States; Martinos Center for Biomedical Imaging, HMS, MGH, Charlestown, MA, United States

**Keywords:** 7T, CBV, layers, somatosensory stimulation, (f)MRI, temporal dynamics

## Abstract

Uncovering the cortical representation of the body has been at the core of human brain mapping for decades, with special attention given to the digits. In the last decade, advances in functional magnetic resonance imaging (fMRI) technologies have opened the possibility of non-invasively unraveling the 3rd dimension of digit representations in humans along cortical layers. In laminar fMRI, it is common to combine the use of highly sensitive blood oxygen level dependent (BOLD) measurements with cerebral blood volume sensitive measurements, such as slice-saturation slab-inversion vascular space occupancy (SS-SI VASO, henceforth referred to as VASO), which are more specific to the underlying neuronal populations. However, the spatial and temporal VASO response characteristics across cortical depth to passive stimulation of the digits are still unknown. Therefore, we characterized hemodynamic responses to vibrotactile stimulation of individual digit tips across cortical depth at 0.75 mm in-plane spatial resolution using BOLD and VASO fMRI at 7T. We could identify digit-specific regions of interest (ROIs) in putative Brodmann area 3b, following the known anatomical organization. In these ROIs, the BOLD response increased toward the cortical surface due to the draining vein effect, while the VASO response was more shifted toward middle cortical layers, likely reflecting bottom-up input from the thalamus, as expected. As an incidental finding, we also saw slightly negative BOLD and VASO responses for non-preferred digits in the ROIs. Therefore, we conducted an exploratory analysis in which we investigated the temporal signal dynamics for BOLD and VASO as a function of distance from activation peaks resulting from stimulation of contralateral digits. With this analysis, we found a triphasic response consisting of an initial peak and a subsequent negative deflection during stimulation, followed by a positive post-stimulus response in BOLD and to some extent in VASO. Similar response dynamics have been observed in the animal literature using invasive methods and in the ipsilateral somatosensory cortex in humans. Furthermore, lateral inhibition has been implicated in models of sensory aging. However, our study is the first to show these temporospatial signal fluctuations directly in human contralateral cortex upon stimulation of individual digits using sub-millimeter BOLD and VASO fMRI. In this context, our findings might offer new windows into the investigations of the potential neuronal excitation–inhibition mechanism in a center-surround architecture in the human somatosensory cortex using layer-specific CBV and BOLD measurements.

## 1 Introduction

Given the importance of our digits in daily life, unsurprisingly, a large portion of the somatosensory system is occupied by their representations. Penfield and Boldrey (1937) and Marshall et al. (1937) were the first to map the somatosensory cortex and investigate digit representations, using invasive methods in humans and macaques, respectively. Over time, the notion arose that there are three distinct body representations in Brodmann areas (BAs) 3b, 1, and 2 on the postcentral gyrus (for a historical overview, see Merzenich et al., 1978). Of the three subregions, BA3b receives the most detailed input from the mechanoreceptors in the fingers via the thalamus and is located on the anterior bank of the postcentral gyrus. Put simply, in BA3b, the fingers are topographically organized with the thumb (D1) being found most inferolaterally, the pinky (D5) most superomedially, and the remaining digits orderly spanning the space in between (Harding-Forrester & Feldman, 2018).

Since the advent of functional magnetic resonance imaging (fMRI) and the discovery of the blood oxygen level dependent (BOLD) contrast (Ogawa et al., 1990), non-invasive investigations in humans were able to replicate the 2 dimensional maps of receptive fields in the digit tips from the seminal papers in animals (Nelson & Chen, 2008; Sanchez-Panchuelo et al., 2010; Schweizer et al., 2008; Steinbach et al., 2024; Stringer et al., 2011; for investigations of within-digit maps, see e.g., Sanchez-Panchuelo et al., 2012, Sánchez-Panchuelo et al., 2014, and Schweisfurth et al., 2014). Furthermore, the quantitative measurements allowed researchers to extend these findings by, for example, exploring structure–function relationships (e.g., differences in cortical thickness between functionally defined sub-regions of the primary somatosensory cortex; Sánchez-Panchuelo et al., 2014).

In the last decade, advances in hardware (most notably ultra-high-field fMRI at or above 7 tesla [T]) have opened the possibility of non-invasively unraveling the 3rd dimension of digit maps—cortical layers. It has been long known from the animal literature that a multitude of processes (e.g., integration of feedforward and feedback processing, predictions, attention) involve cortical layers to different degrees. Therefore, investigating laminar computations is crucial for further understanding somatosensory perception in humans (Dumoulin et al., 2018; Kuehn & Pleger, 2020). Nevertheless, insights into laminar digit representations in BA3b solely using BOLD measurements are sparse (for one example, see Liu et al., 2023). However, Puckett et al. (2017, 2020), Schluppeck et al. (2018), Stoll et al. (2025), and Kalyani et al. (2023) have described the functional BOLD responses in BA3b using sub-millimeter resolution (which is sometimes regarded as a prerequisite for laminar imaging; Huber et al., 2015). The lack of attempts using BOLD to investigate laminar processing in the somatosensory domain may, at least in part, be explained by the fact that the BOLD contrast suffers from the well-known draining vein effect (Turner, 2002). Specifically, changes in blood oxygenation related to neuronal activation are strongest on the venous side of the vascular tree, which transports blood away from the site of activation. Thereby, the resulting BOLD signal is displaced with respect to the active neurons (Olman et al., 2007). At high resolutions, this effect limits the effective spatial specificity of BOLD, despite small voxel sizes (De Martino et al., 2013; Polimeni et al., 2010).

To mitigate the draining vein effect in laminar fMRI, acquiring data using additional non-BOLD contrasts have been proposed, due to their usually higher specificity (for a review, see Huber et al., 2019; alternatively, laminar deconvolution models that are based on assumptions of average vascular physiology (e.g., Marquardt et al., 2018) or vascular calibration methods (e.g., Guidi et al., 2016; Kazan et al., 2017) have been proposed to reduce the draining vein effect in BOLD data). Vascular space occupancy (VASO; Hua et al., 2013; Lu et al., 2003) and more specifically slice-selective slab-inversion (SS-SI) VASO (Huber, 2014; henceforth referred to as VASO) is a promising cerebral blood volume (CBV) sensitive technique due to its comparatively high specificity to the microvasculature and, hence, the underlying neural activity. This way, VASO provides a measure of functional brain changes that is less dominated by large veins than BOLD (Huber et al., 2019). Note however, that the higher specificity of VASO is paralleled by a reduction in detection sensitivity (of sometimes unwanted macrovessels) compared with BOLD. Nevertheless, as the VASO sequence provides both BOLD and CBV-weighted images, it is a tool for non-invasively investigating functional signal changes and neurovascular coupling in humans with relatively high specificity (Huber et al., 2014).

To date, laminar VASO responses in the human primary somatosensory cortex have been described in several papers and conference abstracts (Dresbach et al., 2023; Yang et al., 2019, 2023; Yu et al., 2019, 2021; for VASO studies without laminar results in the somatosensory cortex, see de Oliveira et al., 2023; Huber et al., 2020, 2023). However, these studies investigated responses to stimulation of multiple digits at the same time (e.g. Dresbach et al., 2023), and/or did not investigate the temporal evolution of the responses (e.g., Yang et al., 2019, 2023; Yu et al., 2019, 2021). As a result, the laminar and spatiotemporal characteristics of the VASO response in the human primary somatosensory cortex to the stimulation of digits are still unknown.

To fill this gap, we developed a new sequence protocol that allowed us to acquired depth-resolved BOLD and VASO data covering the entire postcentral sulcus using 7T (f)MRI, while passively stimulating individual finger tips. Furthermore, we tested different experimental protocols in different participants to inform study design and effect sizes for future studies. We expected that, first, our newly developed sequence protocol would provide sufficient signal-to-noise ratio (SNR) to capture laminar BOLD and VASO responses in contralateral human BA3b, second, that the VASO signal would be lower in amplitude, but would show higher microvascular specificity (as shown by an activation peak that is more centered in gray matter) compared with the BOLD signal, and third, that we could characterize VASO and BOLD signal changes across space and time, as far as SNR allows.

## 2 Methods

### 2.1 Participants

Eleven healthy participants (age range: 26–46 years; mean: 31 years, 8 male, 3 female, 7 right handed) with no neurological damage participated in the study after giving written informed consent. The experimental paradigm was approved by the local ethics review committee for psychology and neuroscience (ERCPN) at Maastricht University.

### 2.2 Imaging parameters

**Fig. 1.**
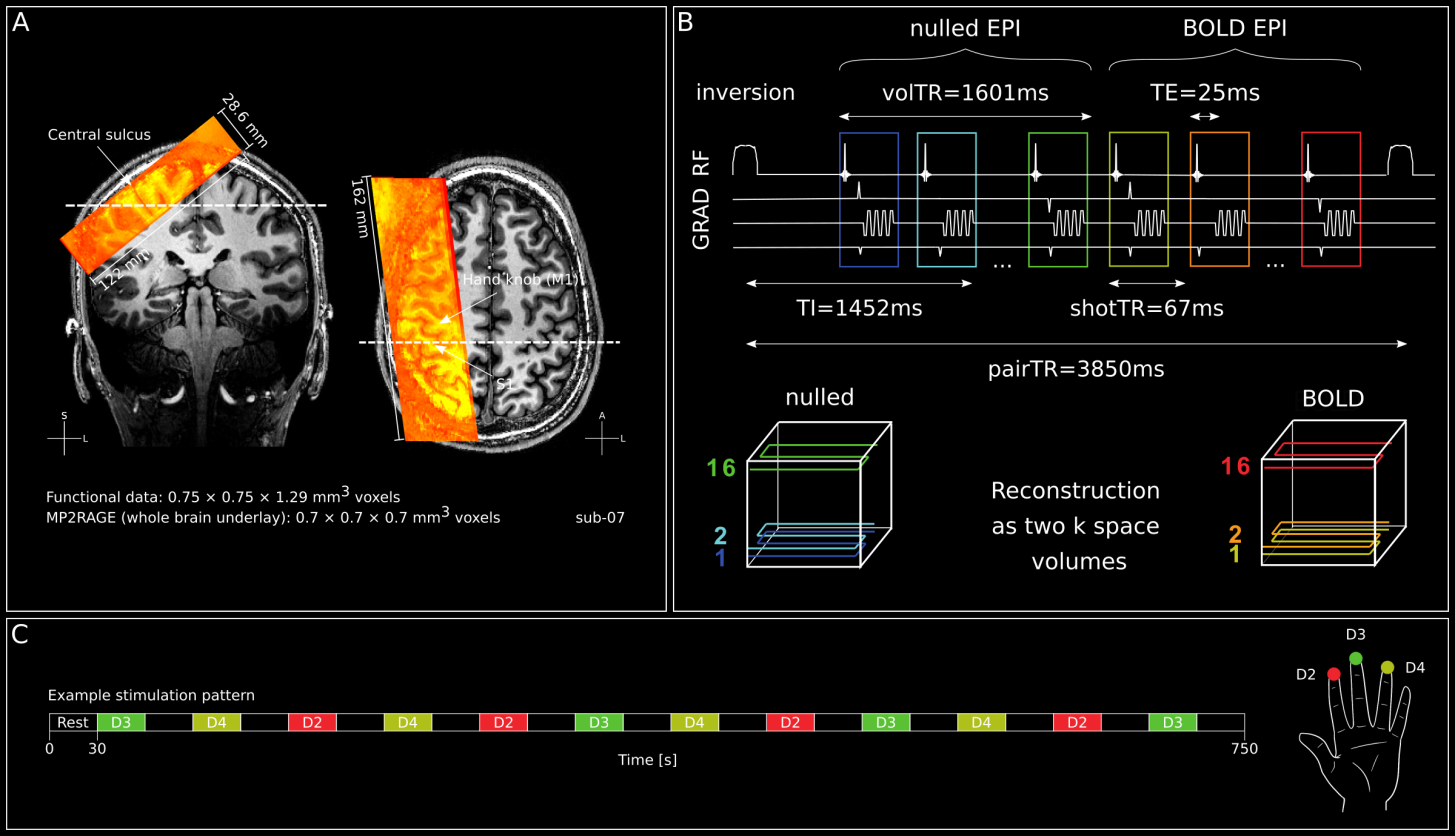
Functional data acquisition and stimulation. (A) Functional EPI data (warm colors) overlaid on MP2RAGE T1-weighted image (T1w, sub-07). The fMRI slab covered the hand area of the right postcentral gyrus (right) and slices were aligned perpendicularly to the central sulcus to maximize resolution through cortical depth in the anterior bank of the postcentral gyrus (left). The location of the respective other slice is indicated by the striped line in each view. (B) Functional EPI data acquisition details giving a schematic overview of relevant imaging parameters. Note: nulled and BOLD images are acquired in an interleaved fashion. Both volumes are later used to derive the “VASO” images. (C) Exemplary stimulation pattern of an entire run. For participants undergoing more than one run of stimulation, we used two different stimulation patterns in an alternating fashion to decrease effects of expectancy. For a full depiction of all stimulation patterns, see Supplementary Figure S1. For a full description of which stimulation pattern(s) were used in each participant, see Supplementary Table S1.

All participants underwent scanning using a 7T Magnetom whole body scanner (Siemens Healthineers, Erlangen, Germany; System version VB17), equipped with a 1-channel Tx, 32-channel Rx head coil (NOVA Medical, Wilmington, DE, USA) at Scannexus (Maastricht, The Netherlands). Functional scans were performed with a 3D-EPI (Poser et al., 2010), SS-SI VASO (Hua et al., 2013; Huber, 2014; Lu et al., 2003) sequence and a nominal spatial resolution of 0.75 × 0.75 × 1.29 mm (22 slices, inversion time [TI] = 1452 ms, effective time of repetition [pairTR] = 3850 ms, volume acquisition time [volTR] = 1601 ms, time between excitation pulses in 3D readout module [shotTR] = 67 ms, echo time [TE] = 25 ms, partial Fourier factor = 6/8 using projection onto convex sets [POCS; Nakamura et al., 2016] reconstruction with 8 iterations, FLASH GRAPPA 3 [Talagala et al., 2016], varying flip angle [FA] between 26 and 80°, read bandwidth = 1064 Hz/Px, 1st phase encoding direction along anterior-posterior axis, field of view [FOV] = 162 mm and 122 mm in read and [1st] phase encoding directions respectively). We used the vendor-provided “improved GRAPPA WS” algorithm with at least 1000-fold overregularization and small GRAPPA kernels of 2 × 3 (phase × read). Slice position and orientation were chosen individually for each participant. Specifically, the FOV covered the entire postcentral gyrus without fold-over artifacts, while optimizing resolution perpendicular to its anterior bank (see Fig. 1A; for a study using a similar approach in the primary motor cortex, see Huber et al., 2020).

Two types of anatomical images were acquired. First, whole-brain images (0.7 mm isotropic, 240 slices, TI1 = 900 ms, TI2 = 2750 ms, TR = 5000 ms, TE = 2.47 ms, FA1/FA2 = 5°/3°, bandwidth = 250 Hz/Px, GRAPPA acceleration factor = 3, FoV = 224 × 224 mm) were acquired using MP2RAGE (Marques et al., 2010). Second, high-resolution anatomical images (0.5 mm isotropic, 60 slices, TI1 = 900 ms, TI2 = 2750 ms, TR = 6000 ms, TE = 4.02 ms, FA1 = 6°, FA2 = 7°, bandwidth = 140 Hz/Px, acceleration factor = 2, FOV = 160 × 160 mm) were acquired with the same slab orientation as the functional data, also using MP2RAGE (Marques et al., 2010). A complete set of the scan protocols is available on: https://github.com/sdres/sequences/tree/master/S1_templates and the sequences are available for VB17B-UHF via SIEMENS C2P.

### 2.3 Experimental procedure

During the stimulation runs, three digit tips of the left hand (index-, middle-, and ring-finger, in the following referred to as D2, D3 and D4, respectively) were stimulated by means of piezoelectric vibrotactile stimulators (Supplementary Fig. S1A; mini PTS system, Dancer Design, UK). The stimulation setup was controlled in Presentation® software (Version 16.0, Neurobehavioral Systems, Inc., Berkeley, CA, www.neurobs.com) and the corresponding script is available on: https://github.com/sdres/s1Anfunco/blob/main/code/stimulation.

In each run, the digit tips were stimulated at 25 Hz, using a 30s on–off block design. Per block, an individual digit was stimulated, while the stimulation order of digits across blocks was pseudo-randomized (for an example stimulation order across blocks within a run, see Fig. 1C). To reduce habituation effects within a block of stimulation, we interspersed the stimulation with short interruptions of 100 ms, as done in previous studies (e.g., Besle et al., 2013). For each participant, we acquired 1–3 runs of stimulation, resulting in 4–12 repetitions/digit. For one participant (sub-12), we also stimulated the thumb (D1) and little finger (D5) of the left hand in addition to D2–D4. However, in the analyses for this manuscript, we only used the results from the stimulation of D2–D4. For a complete description of how many repetitions were acquired, how many digits were stimulated and which stimulation pattern(s) was (were) used per participant, see Supplementary Table S1.

Finally, resting-state data were acquired for all participants. However, the results of the resting-state analysis are beyond the scope of this manuscript and will not be discussed further.

### 2.4 Anatomical data processing

The high-resolution anatomical MP2RAGE T1w images (0.5 mm isotropic) were co-registered using rigid transformations in ANTsPy (Avants et al., 2011; v0.3.3s) and subsequently averaged. Signal differences due to inhomogeneities of the radio-frequency field were corrected using *n4_bias_field_correction* as implemented in ANTsPy. To limit computing demands, we reduced our anatomical data to a visually defined box around the postcentral gyrus for each participant individually. After this data reduction, we spatially upsampled the data with a factor of 3, resulting in a nominal voxel size of ∼0.16 mm isotropic.

To estimate gray matter borders, an initial tissue segmentation was performed using standard parameters in FSL FAST (Zhang et al., 2001; v6.0.5). Crucially, the resulting segmentations were manually improved using ITK-SNAP (Yushkevich et al., 2006; v3.8.0) by an expert (author S.D.) and quality controlled by another expert (author O.F.G.). Special care was given to the region on the postcentral gyrus opposite of the “hand-knob” of the primary motor cortex.

## 3 Functional Data Processing

### 3.1 Pre-processing

Initially, the concomitantly acquired nulled (CBV-weighted, with BOLD contamination) and not-nulled (henceforth referred to as BOLD) time series of each run were separated and further processed individually. In order to remove time points in which a steady state was not reached yet, we replaced the first, second, and third volumes with the fourth, fifth, and sixth volumes, respectively. In the following, motion correction was performed using ANTsPy (Avants et al., 2011; v0.3.3s). Specifically, time courses were corrected for within-run motion by aligning each volume to the mean of the first three volumes, using rigid transformations and second order spline interpolation. Participant motion is often regarded a problem in high-resolution fMRI. Therefore, we computed framewise displacements (FDs) within each run (Power et al., 2014) and computed motion outliers (as defined in the program *fsl_motion_outliers*) to assess the severity of participant motion. A summary of motion exceeding the voxel size and motion outliers is shown in Supplementary Figure S2. In brief, a total of 9198 volumes (nulled and not nulled combined) were acquired across participants. Of those, 30 volumes (<0.33%) showed FDs greater than our in-plane voxel resolution (0.75 mm), with only a single volume with an FD of >2 mm. Given this low number of affected volumes, we did not exclude any data based on participant motion.

Thanks to the inherent T1 contrast due to the inversion recovery nature of the VASO sequence, we could derive run-wise T1w images in EPI space (T1w-EPI) by computing the voxel-wise inverse variation coefficient of the concatenated nulled and BOLD time courses. The T1w-EPIs of all stimulation runs were then co-registered to the T1w-EPI of the resting-state run. Thereafter, we reapplied the motion correction for all stimulation runs while concatenating the transformation matrices from the within- and between-run registrations. Finally, we computed a T1w-EPI across all runs to maximize the SNR for the registration with the anatomical MP2RAGE data (see section on region of interest [ROI] definition).

To limit computing demands, we also reduced our functional data to a visually defined box around the postcentral gyrus for each participant individually. After that, time courses were temporally upsampled by a factor of 2 using AFNI’s *3dUpsample* (Cox, 1996; 7th order polynomial interpolation) and the first volume of the nulled time course was duplicated in order to temporally match the nulled and BOLD data. Note that the data in the present manuscript were collected using the VASO version for Siemens software platform VB17. For VASO versions on the VE software platform, the first BOLD volume has to be duplicated in order to temporally match the BOLD and nulled time courses. Finally, residual BOLD contamination of the nulled time course was corrected by dividing the nulled by the BOLD time course. The BOLD-corrected nulled time courses are henceforth called VASO.

### 3.2 (Statistical) response estimation

To estimate spatial stimulus-evoked VASO and BOLD responses, we conducted a general linear model (GLM) analysis in FSL (v6.05.2; Woolrich et al., 2001). Here, we used individual predictors for the stimulation of the three digits (five digits for sub-12, see Supplementary Table S1) convolved with a standard hemodynamic response without temporal derivative (mean lag: 6 seconds, std. dev: 3 seconds), while applying a high-pass filter (cutoff = 0.01 Hz) and no additional smoothing. Furthermore, we used the output of *fsl_motion_outliers* from FSL and six motion regressors (three rotations and translations) from the motion correction as additional confounds to minimize residual influences of head motion (Power et al., 2014). For participants who underwent more than one stimulation run, z-maps from the GLM were combined across all runs using a fixed-effects analysis.

**Fig. 2.**
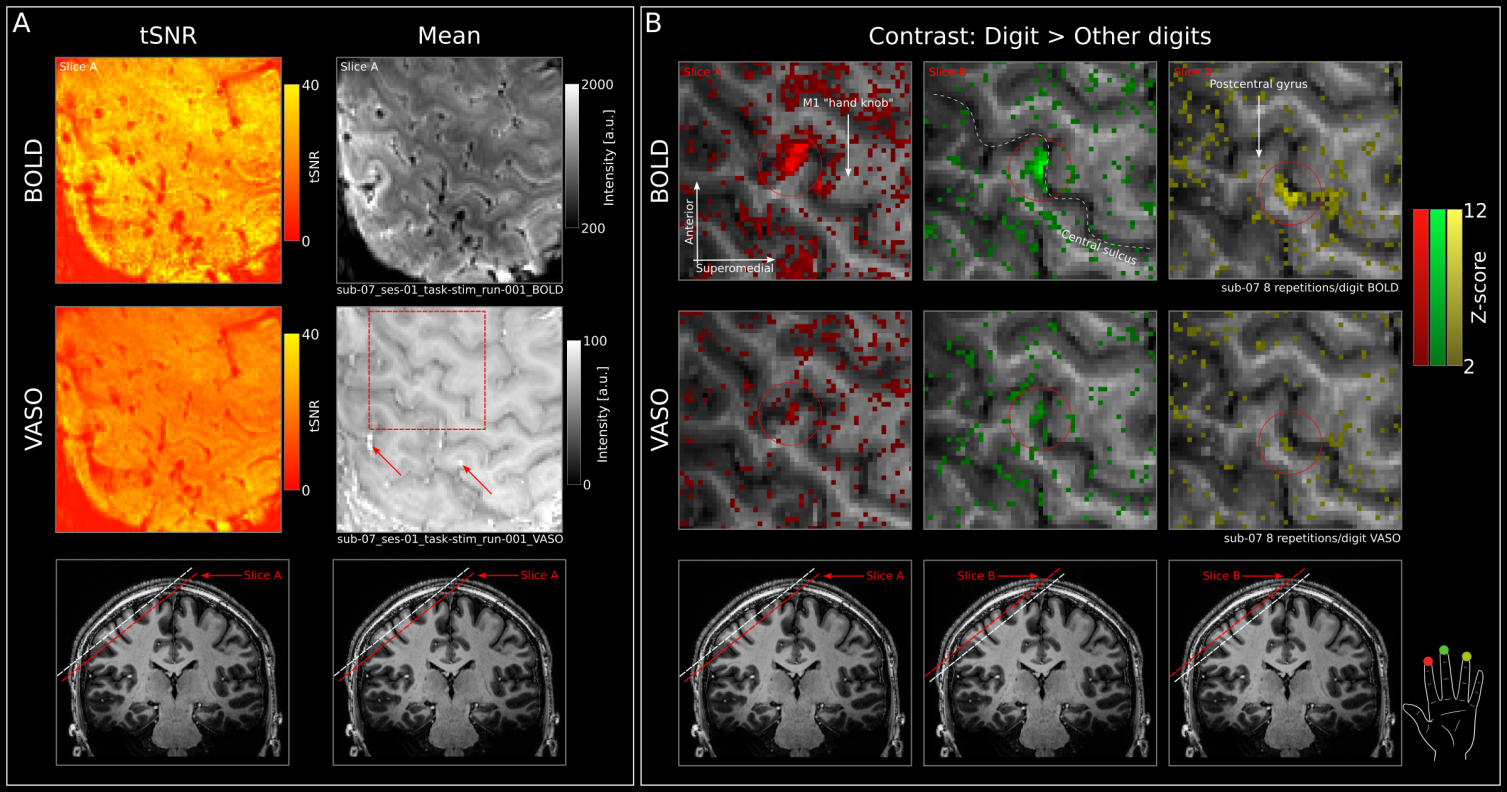
Quality assessment and stimulation results. (A) BOLD (upper row) and VASO (middle row) tSNR (left) and mean (right) maps of an individual run of a representative participant (sub-07). Red square in the middle right panel indicates the zoomed-in section in B, while the slice position with respect to the entire brain is indicated in the lowest row. Mean BOLD and VASO images are without major artifacts that are common in high-resolution fMRI using VASO (e.g., “fuzzy ripples”; Huber et al., 2025). Bright voxels in the mean VASO image may indicate inflow of non-inverted blood (red arrows) but are outside the ROI and can, therefore, be ignored. (B) BOLD (upper row) and VASO (middle row) stimulation results of an individual participant (sub-07) showing the statistical z-map (threshold: z > 2) resulting from a GLM analysis in which we contrasted each finger against all other fingers overlaid on a T1w image directly in EPI space (therefore, without issues regarding alignment between functional activation and anatomical reference). Note that we display only individual contrasts within a given map for better visual clarity. In addition, note that we did not apply thresholds based on cluster size, to give a better feeling for the noise characteristics in the data.

For visual inspection and display purposes of the statistical maps resulting from the GLM, the range of z-values of the BOLD and VASO activation maps is kept between 4–12 and 2–6, respectively, in all participants (Fig. 2; Supplementary Figs. S3–S5). This way, the difference in activation strength and noise level across participants is visible more clearly, which gives a better feeling for the data quality. For reference, a z-threshold of 4 relates to a p-value <0.001 (uncorrected), while a z-threshold of 2 relates to a p-value <0.05 (uncorrected). The different z-thresholds for BOLD and VASO were chosen to account for the difference in detection sensitivity between the two contrasts.

### 3.3 Definition of digit ROIs

**Fig. 3.**
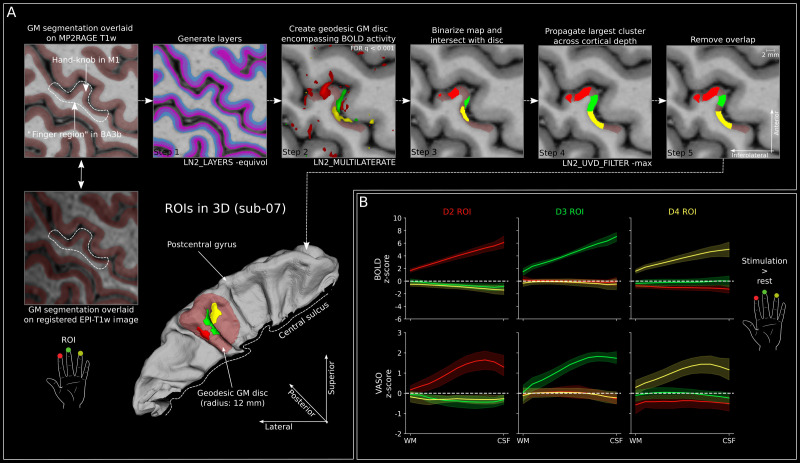
ROI generation and activation across cortical depth. (A) Leftmost column shows the gray matter segmentation overlaid on the upsampled MP2RAGE T1w image (nominal resolution: 0.16 × 0.16 × 0.16 mm^3^ isotropic; upper row) and the same segmentation overlaid on the registered EPI-T1w image (lower). Note that the segmentation matches the gray matter ribbon in both panels, especially in the area of interest, highlighting proper registration. Dashed lines indicate the approximate region in which the three fingers of interest are represented in this slice. The remaining tiles show the steps in the automatic process of generating digit-specific ROIs based on BOLD activation. Note that in step 1, we display three layers for simplicity. However, to plot layer profiles (3B, Supplementary Figs. S7–S8), we extracted z-scores from 11 layers, whereas to plot depth-dependent time courses, we extracted event-related averages from 3 layers. Also note that in step 2, statistical significance of voxel activation was estimated using a false discovery rate (FDR) threshold with q > 0.001. Importantly, we used the GLM contrast “digit > other digits” (e.g., for BOLD D2: [D2: +1; D3: -0.5; D4: -0.5]), which is similar to a winner-takes-all approach. This is important because a given voxel in the somatosensory cortex may respond to stimulation of multiple digits. Furthermore, ROI generation was performed for each digit independently in steps 2–4. Therefore, a given voxel may still have been included in multiple digit ROIs at the end of step 4. If this was the case, this voxel was removed from all digit ROIs (step 5). This automatic process resulted in ROIs spanning multiple slices in all participants (shown here in 3D for one representative participant [sub-07]). (B) Stimulation-induced activity (z-scores) across cortical depth for BOLD (upper row, n = 11) and VASO (lower row, n = 11) for each digit ROI separately (GLM contrast: digit > rest). Values were extracted from participant-specific ROIs (example depicted for sub-07 in **A** using 11 depth levels. Z-scores across cortical depth clearly show strongest BOLD activity toward the cortical surface and response peaks between middle and superficial cortical layers for VASO in response to stimulation of the preferred digit within each ROI. White dashed line indicates 0 line and shaded area indicates 95% confidence interval across participants.

To quantify activation across cortical depths, we semi-automatically generated digit-specific ROIs. Figure 3A shows the procedure and resulting ROIs in 3D for a representative participant (sub-07).

First, the functional data were registered to the upsampled high-resolution MP2RAGE T1w images. This was done using the T1w-EPI described above. To guide the automatic registration process, we first manually aligned the T1w-EPI images to the MP2RAGE T1w images in ITK-SNAP and saved the transformation matrix. This initial transformation was then refined using non-linear SyN transformation and fifth order B-spline interpolation using *AntsRegistration*. Non-linear registration was used to account for the geometric distortions in the functional EPI data. Furthermore, we used a registration mask encompassing the area of digit representations opposite the hand knob on the postcentral gyrus in which the optimization metric (cross correlation) should be optimized. Figure 3A (leftmost column) shows the gray matter segmentation overlaid on the MP2RAGE T1w image and the registered T1-EPI. It can be seen that the segmentation accurately follows the gray matter ribbon in both images, indicating the high registration accuracy. Finally, the functional activation maps were registered using the transformation matrices estimated above.

The ROIs were then generated in the upsampled MP2RAGE T1w space using the following five-step procedure:

1. From the segmentation (see section on anatomical data processing), we computed the normalized cortical depth of each gray matter voxel using *LN2_LAYERS* with the equivolume option as implemented in LayNii (v2.6.0).
2. We then generated a participant-specific geodesic gray matter disk using *LN2_MULTILATERATE* with a radius of 12 mm to limit the ROI generation to the digit region on the anterior bank of the postcentral gyrus (red opaque disk in Fig. 3A). The central point of the disk was chosen individually for each participant so that the disk encompassed the largest significantly active BOLD activation clusters for all three digits (for central points of individual participants, see Supplementary Table S2). Voxels were deemed “active” if they exceeded the FDR threshold (q = 0.001; contrast digit > other digits). For the depiction of the geodesic disk and the ROIs in all participants, see Supplementary Figure S6. Here, it can be seen that for most participants, the ROIs do not extend until the edge of the geodesic disk (radius 12 mm), thus showing that we were able to capture the entirety of the largest activation cluster of each digit in BA3b. For one participant (sub-14) however, this was not possible without further extending the radius of the disk. Here, the largest activation cluster for D2 extended far into putative BA1 on the crown of the postcentral gyrus. Therefore, in this participant, we chose a central point for the disk that would cut off the largest D2 cluster around the transition between the anterior bank of the postcentral gyrus and its crown (see Fig. S6F).
3. The FDR-thresholded (q = 0.001) BOLD activation map of each digit, resulting from the contrast digit > other digits, was binarized and limited to cortical gray matter by intersecting the binary digit mask with the gray matter disk (generated in step 2).
4. We used the cortical parametrization from the *LN2_MULTILATERATE* program and the binary digit masks (generated in step 3) to propagate the largest cluster of the thresholded and binarized digit masks across cortical depth using *LN2_UVD_FILTER* with the -max option (Pizzuti et al., 2023). Briefly, the algorithm iteratively places a cylinder (chosen radius: 0.45 mm) around each voxel within the gray matter disk perpendicular to the cortex and gives all voxels within the cylinder the maximum value found within it. Given that we used the binarized digit map as input, voxels within cylinders containing at least one “active” voxel are labeled 1, whereas voxels within other cylinders are labeled 0. With this procedure, we assured that a similar number of voxels was included across depth levels. Importantly, this was done for each digit independently.
5. Finally, we removed any voxels from the three digit ROIs that were part of multiple digit ROIs. Note that the digit ROIs were created independently for each digit using the maps resulting from the contrast digit > other digits. Therefore, the thresholded and binarized activation maps show little to no overlap. However, there are four cases in which our procedure may label a given voxel as belonging to multiple digit ROIs. First, a given voxel may be part of the thresholded and binarized activation map for two digits if it shows a similarly positive response to stimulation of those digits, while the response to the third digit is very negative. This case appears in at least one voxel in 4 of 11 participants. However, the number of voxels that are part of the thresholded and binarized maps of two digits is negligible (max. 0.7% of the voxels that were part of the thresholded and binarized maps). Second, overlap between digit ROIs may occur in regions where digit-selectivity switches from one digit to another. This is because the cylindrical kernel of step 3 of the ROI generation process has a radius of 0.45 mm and the propagation across cortical depth was performed on each digit map individually and at a nominal resolution of 0.16 mm isotropic. As a result, if two neighboring voxels are part of different digit maps, they might be included in the cylinder of the respective other map and, therefore, labeled as belonging to both digit ROIs. Third, digit preference may change across cortical depth. For example, some cylinders may contain voxels preferring one digit in superficial layers, whereas voxels at lower cortical depths respond more strongly to stimulation of another digit. Consequently, all voxels within the cylinder will be labeled as belonging to both digit ROIs (For an example of this case, see Fig. 3A steps 3–5). Finally, a given voxel may be labeled as belonging to two-digit ROIs in regions with high curvature. Specifically, in a gyrus, a given voxel close to the gray–white matter border may be included in the cylinders placed around multiple voxels closer to the cortical surface due to the local curvature. Therefore, if the digit preference of these voxels at the cortical surface is different, the deep-layer voxel will be part of multiple digit ROIs. However, the reverse scenario is true at the fundus of a sulcus. For these reasons, we decided to remove all voxels from all digit ROIs that were labeled as belonging to more than one digit ROI. The final digit ROIs had an average extent of 58.54, 58.76, and 44.38 mm^3^ across participants for D2, D3, and D4, respectively (for digit-specific ROI volume of individual participants, see Supplementary Fig. S6K).

### 3.4 Extraction of event-related averages

In order to analyze the temporal evolution of activation across cortical depths, we extracted event-related averages from three cortical depth levels and three digit ROIs for BOLD and VASO independently. The process involved four steps:

1. We first registered each functional volume of all stimulation runs to the upsampled MP2RAGE T1w image using the linear and non-linear registration transforms estimated before (see “functional data processing” section) and limited the data to the geodesic gray matter disk (see “definition of digit ROIs” sections).
2. We then linearly detrended the voxel time courses, regressed out the motion traces and motion outliers, and computed signal changes (%) with respect to the mean of the original time series using the program *signal.clean* implemented in nilearn (v0.10.3; Abraham et al., 2014).
3. Then, we averaged the time series for each of the three-layer compartments within each of the three digit ROIs for each run, resulting in a nine (three layer compartments × three ROIs) × n matrix per run, where n is the number of volumes in the run. The three layer bins were generated by binning the normalized cortical depth metric given by LN2_LAYERS (see section on ROI definition, step 1).
4. Finally, for each stimulation trial per run, we extracted signal changes from 15 volumes before stimulation onset up until 70 volumes after stimulation onset for each ROI and layer compartment separately.

### 3.5 Definition of distance ROIs

To investigate the response characteristics as a function of distance from activation peaks, we first identified the voxel with the highest z-score in response to the stimulation within gray matter of each digit individually. We then defined seven Euclidean distance bins within gray matter ranging from 0–2 to 12–14 mm (in 2 mm increments) from the peak BOLD activation for D2, D3, and D4 independently (Fig. 5A) using *LN2_GEODISTANCE* implemented in LayNii (v2.6.0). The area in which we defined the distance bins was limited to the geodesic disk used for the creation of ROIs (red shaded disk in Fig. 3A) because the tissue segmentation was performed especially carefully here.

The analysis code is available online at https://github.com/sdres/s1Anfunco.

## 4 Results

### 4.1 Quality assessment

Figure 2A shows the mean images and temporal SNR (tSNR) maps for BOLD and VASO in a representative participant (sub-07). Crucially, the mean images are without major artifacts that are common in high-resolution fMRI using VASO. Potential artifacts include blurriness due to motion, inflow of not-inverted blood, geometrical distortions, or ghosts that show up as dark spots in the images (including “fuzzy ripples”; Huber et al., 2025). While we found indications of inflow effects (red arrows on the mean VASO image in Fig. 2A), the affected voxels were outside of the ROI and, therefore, do not influence the results discussed below. Additionally, the mean VASO images show a reasonable gray matter to white matter contrast due to the longer pairTRs used here (compared with, e.g., Dresbach et al., 2023). Finally, the tSNR maps show higher values for BOLD than VASO, as expected. Nevertheless, both contrasts show a tSNR >10 which is adequate for fMRI at sub-millimeter resolutions (Huber, 2020). For a quantification of tSNR values in the postcentral gyrus on the group level and for all participants separately, see Supplementary Figure S2A.

### 4.2 Visual inspection shows expected digit organization in putative BA3b

In this section, we first anecdotally describe the BOLD and VASO results from visual inspection of the activation maps, whereas we quantify the results across cortical depths based on the objective ROI definition in the following section. Note that for visual inspection and display purposes of the statistical maps resulting from the GLM, the range of z-values of the BOLD and VASO activation maps was kept between 2 and 12 in all participants (Fig. 2; Supplementary Figs. S3–S5). This way, the difference in activation strength and noise level across participants is visible more clearly, which gives a better feeling for the data quality. For reference, a z-threshold of 2 relates to a p-value <0.05 (uncorrected).

Upon visual inspection, tactile stimulation of individual digit tips (D2–D4) of the left hand evoked strong (p < 0.05 [uncorrected]) BOLD responses in the right primary somatosensory cortex of all participants (GLM contrast: respective digit > all other digits; e.g., for D2 in BOLD [D2: 1, D3: -0.5, D4: -0.5]). The BOLD results of one representative participant (sub-07) are shown in Figure 2B (upper row), whereas the results from the remaining participants are shown in Supplementary Figures S3–S5. We observed stimulation-induced activation in the putative BA3b on the anterior bank of the postcentral gyrus, following the known topographical organization in 10 out of 11 participants. Specifically, we found D2–D4 to be represented in order from lateral to medial and inferior to superior locations. Note that the data were acquired with an oblique slab orientation in all participants. Therefore, even when multiple finger representations are seen in the same slice, more medial representations are also more superior.

In the deviating participant (sub-10), we found two clusters for D3. One cluster is located laterally and inferior to D2. The other cluster is located between D2 and D4, as would be expected. Importantly, the “irregular” cluster was found to be larger and showed stronger activation within gray matter. Furthermore, similarly irregular patterns of digit representations have been observed in this participant with fMRI before (Steinbach et al., 2023) and have been observed in monkey electrophysiology and human fMRI studies (Merzenich et al., 1987; Sanchez-Panchuelo et al., 2012). Therefore, we considered the “irregular” cluster to be the representation of choice in further analyses.

In the VASO data, clearly identifiable responses (p < 0.05 [uncorrected]) to the stimulation of all digits can be seen in 7 out of 11 participants (sub-06, sub-07, sub-09, sub-12, sub-14, sub-15, sub-18). Figure 2B (middle row) shows the VASO results of one participant (sub-07; for the data of other individual participants, see Supplementary Figs. S3–S5). In a further two participants (sub-16, sub-17), responses were clearly visible for two out of three digit representations. For the remaining digit representation, it was difficult to find active clusters in the noisy activation maps, however, clusters with increased activation in response to stimulation could be identified in gray matter close to where also BOLD activation was located. In the two remaining participants (sub-05 and sub-10), one digit representations could be identified in VASO when taking the BOLD activation into account in a similar way. The other two representations could not clearly be picked up from the noise in the data.

### 4.3 Stimulation shows expected activation across cortical depth

The group-level BOLD and VASO responses across cortical depth within the digit ROIs are shown in Figure 3B (for data from individual participants, see Supplementary Figures S7 and S8). For each ROI, z-scores resulting from finger stimulation of each finger were extracted using 11 depth levels and averaged across participants (GLM contrast digit > rest). Unsurprisingly, we found largest activation for the preferred digit in all digit ROIs. Furthermore, the BOLD activity clearly showed an increase in activation toward the cortical surface for the preferred digit within each ROI, as expected based on the draining vein effect. VASO activity shows peaks more toward middle cortical layers and a decrease toward the surface for the preferred digit in each digit ROI. Note that the ROIs in Figure 3A were based on the BOLD activation, which has a larger extent than the VASO activation due to its higher sensitivity (also reflected in higher z-scores in Figs. 2B and 3B). As a result, the ROIs include many voxels without substantial VASO activity and likely underestimate the sensitivity and specificity of the extracted VASO activation.

Finally, we find slightly reduced negative responses for other digits within the D2 and D4 ROIs for both BOLD and VASO (activity below dashed lines in Fig. 3B). Interestingly, activity for digit representations that are further away is more negative in most cases (except for VASO in the D2 ROI). In line with this, there is only very weak negative activity within the D3 ROI occurring with stimulation of D2 or D4, given its position centered between the representations of D2 and D4. Furthermore, the negative response seems to be strongest in superficial layers for both BOLD and VASO. However, this effect is rather small and it should be interpreted with caution based on the z-score profiles alone.

## 5 Temporal Analysis

### 5.1 Event-related averages show a triphasic response to distant, non-preferred digits

**Fig. 4.**
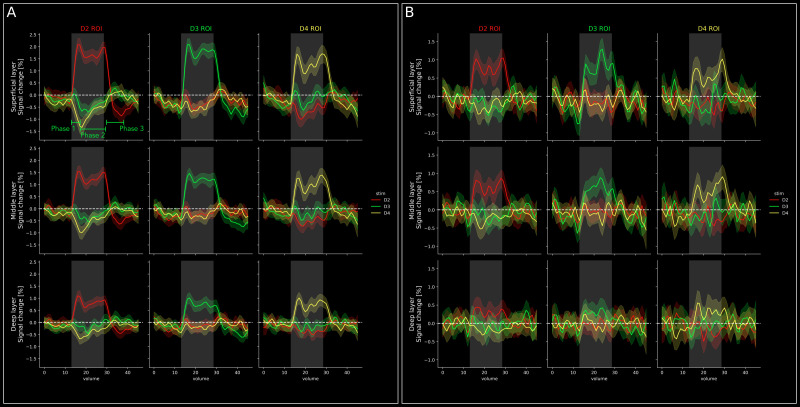
Event-related averages across cortical depths. (A) Event-related averages for BOLD data (n = 11) in superficial (top) middle (middle) and deep (bottom) layer compartments. Gray-shaded area indicates the stimulation period. The striped line indicates the baseline, which was calculated as the voxel-wise mean of the entire run. The shaded area around lines signifies the 95% confidence interval across trials. Note the strong responses during stimulation of the preferred digit and indications of a triphasic response to non-preferred digits in all ROIs. The three phases of this triphasic response (phase 1: initial peak, phase 2: trough, phase 3: post-stimulus peak) are indicated for the response to D3 (green line) in superficial layers of the D2 ROI. (B) Same as **A**, but for VASO data (n = 11). Similar activation patterns can be observed, however, with higher noise.

In a GLM analysis, a certain hemodynamic response function is assumed, which may bias the results against response characteristics that do not follow the expected hemodynamic response shape. Therefore, we investigated temporal response characteristics in a model-free approach. The resulting group-level event-related averages for BOLD and VASO are shown in Figure 4A and B, respectively.

As expected based on our ROI definition (digit > other digits), we found sustained BOLD responses to stimulation of each digit to be highest in the corresponding ROI. Furthermore, we found indications of a triphasic response to non-preferred digits in BOLD. Specifically, we found a small initial BOLD signal increase upon start of stimulation of a non-preferred digit (phase 1), followed by sustained negative signal changes during the stimulation of non-preferred digits (phase 2) and a small positive response after the cessation of stimulation of non-preferred digits (phase 3). The negativity (phase 2) is visible in all ROIs, however, with varying magnitude. Specifically, the negativity is largest during stimulation of non-preferred digits that are further away (e.g., it is larger in the D2 ROI during stimulation of D4 than D3), and it is larger in superficial than in deeper layers (Fig. 4A). While the initial increase did not necessarily reach positive signal changes, it constituted a consistent increase with respect to previous time points.

For VASO, we also found sustained responses to stimulation of the preferred digit in the corresponding ROI (Fig. 4B). Furthermore, response amplitudes to stimulation of the preferred digits were comparable in superficial and middle layers with slightly higher responses in the superficial layer. Smallest responses were seen in the deep layer. For the VASO z-scores across cortical depth, we observed a slight signal decrease toward cortical surface (see previous section; Fig. 3B). The fact that we did not observe a similar effect in the event-related averages can be explained by larger amounts of partial voluming due to signal extraction from fewer layers (11 layers for z-score profiles vs. 3 layers for event-related averages). Furthermore, z-scoring will reduce the signal of superficial layers due to the higher noise level, which is not the case in the raw % signal change. Finally, we also observed slightly decreased VASO activation in response to stimulation of non-preferred digits, however, to a lesser extent in BOLD. However, the initial and post-stimulus peaks are not visible in the VASO responses from the ROIs due to the higher noise level compared with BOLD.

Note that the inter-stimulus interval in our experiment was 30 seconds. Therefore, the volumes 0–15 and volumes 30–45 in Figure 4 were actually overlapping. In volumes 30–45, we can see a signal decrease for non-preferred digits and remnants of an undershoot in preferred digits. Therefore, the fact that the initial response to non-preferred digits did not exceed 0 is likely due to a carryover effect from preceding trials. However, while the data come from the same time points, they are still independent because the same digit was never stimulated multiple times in a row.

### 5.2 The triphasic response changes as a function of distance from the activation peak

**Fig. 5.**
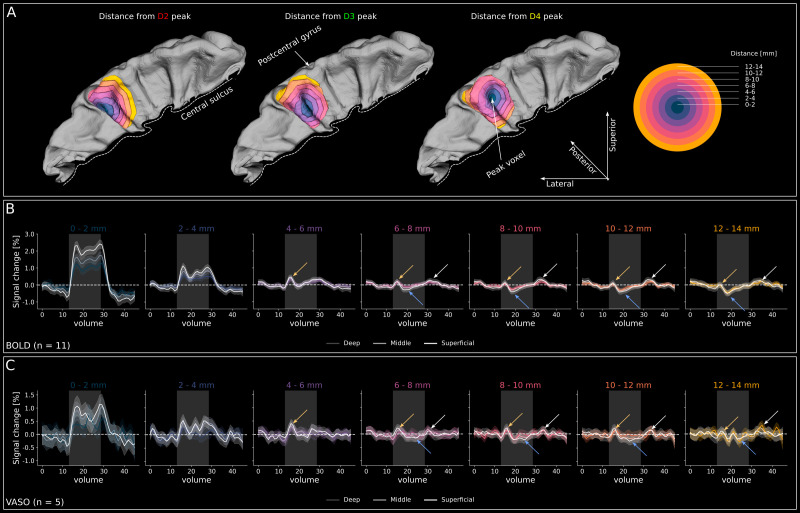
Triphasic response as a function of distance from peak activation. (A) 3D representations of the postcentral gyrus with Euclidean distance bins from the BOLD peak voxel of D2, D3, and D4 response (from left to right). The participant (sub-07) is the same as in Figs. 2 and 3). The color code of distance rings is explained on the right. (B) Group-level (n = 11) BOLD event-related averages for seven distance bins with respect to the peak voxel, collapsed over all three stimulated digits. The data are plotted for superficial, middle, and deep layers per distance bin. Superficial layers are white in all panels, whereas middle and deep layers are color coded with increasingly darker colors of the respective distance bin (Fig. 5A). The yellow arrows indicate the small initial signal increase upon stimulation. The cyan arrows indicate the following signal decrease. The white arrows indicate post-stimulus signal increase in more distant bins. Gray-shaded area illustrates the stimulation period. Shaded area around lines signifies the 95% confidence interval across trials. (C) Same as **B**, but for the VASO data of the subset of participants that showed indications of a triphasic response on the individual participant level (n = 5; see Supplementary Figs. S9 and S10).

To further investigate the distance dependence of the triphasic response, we plotted group-level (n = 11) BOLD signal changes in superficial, middle, and deep layers as a function of distance from the voxel within gray matter that showed the highest BOLD z-score in response to stimulation of each individual digit. The distance ROIs of the three stimulated digits of one representative participant (sub-07) are shown in Figure 5A and the group-level BOLD signal changes across the distance rings collapsed over the three digits are shown in Figure 5B. Crucially, and unlike in Figure 4, the voxel selection in this analysis is almost completely independent from a GLM contrast (except for the definition of the peak voxel) but depends on anatomical distance from a peak voxel. Therefore, the effects cannot be explained by a voxel selection based on response characteristics.

For the 0–2 mm bin, the response shape resembles the combined responses from the digit ROIs (compare with Fig. 4A). Similarly, for the 2–4 mm bin, we found activation resembling the expected response pattern from regions responding to the stimulation of preferred digits, albeit weaker. The 4–6 mm distance bin starts to show a small initial positive response (yellow arrows in Fig. 5B), followed by a slight signal decrease below baseline (cyan arrows in Fig. 5B). In the remaining distance bins, the initial BOLD response seems to become increasingly weaker, whereas the following negative signal changes seem to become increasingly negative. Finally, the more distant distance bins (>6–8 mm) show a notable positive post-stimulus peak starting at the stimulus offset (white arrow in Fig. 5B).

To investigate the evolution of timing and magnitude across phases of the triphasic response, we quantified the BOLD times to peak (TTP) and the respective BOLD signal changes (%) of the initial peak, trough, and post-stimulus peak for distance bins >4–6 mm. The initial peak was defined as the highest deflection within the stimulation period, while the trough was defined as the lowest deflection after the peak and before the end of the stimulation period. The post-stimulus peak was defined as the highest deflection in a window of eight volumes (∼15.4 seconds) after stimulus offset. For the post-stimulus peak, we only took distances >6–8 mm into account, because we did not observe a corresponding response in the group results for smaller distances (Fig. 5B). Crucially, because of the high variability across participants, we extracted information on BOLD and VASO TTPs and respective signal changes based on the mean response across participants.

**Fig. 6.**
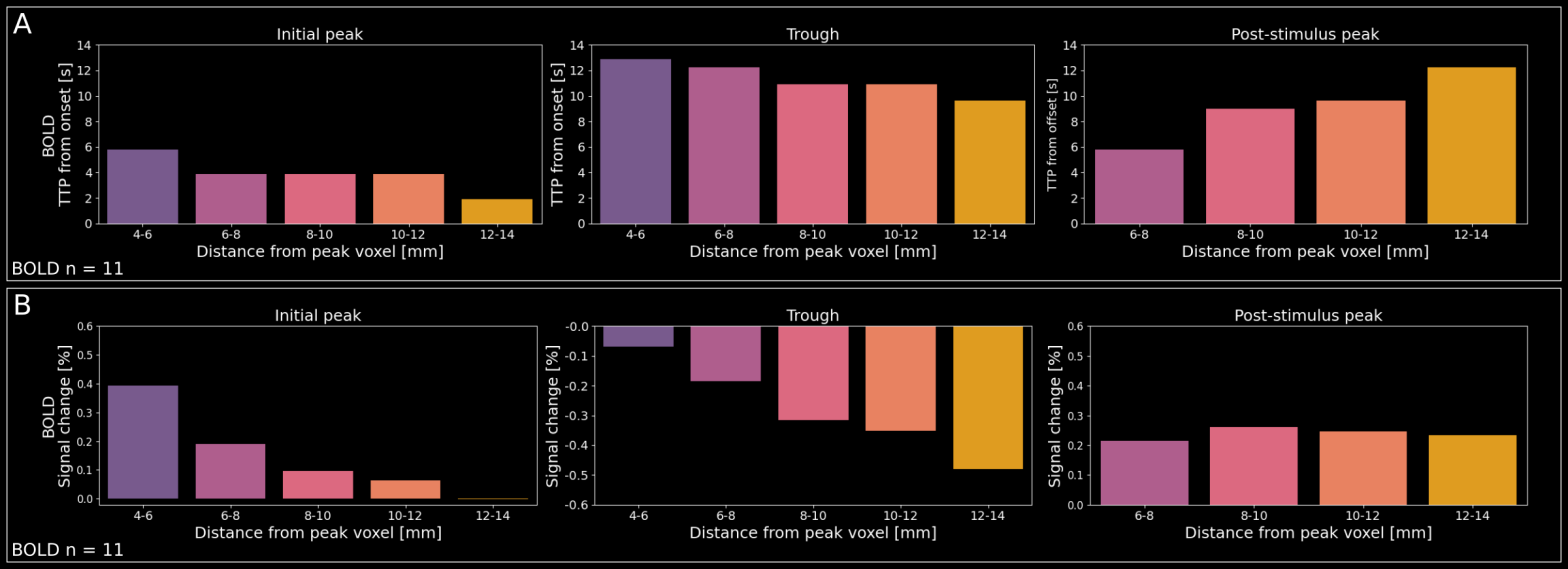
Timing and amplitude of the triphasic response for each phase independently. (A) BOLD (n = 11) times to peak (TTP) of the initial peak (left), trough (middle), and post-stimulus peak (right) of the triphasic response for distance bins ≥4–6 mm. Note that for the post-stimulus peak, we only took distances ≥6–8 mm into account because for smaller distances, we did not observe a corresponding response feature in the group results. For the initial peak and trough, times were taken with respect to stimulus onset. For the post-stimulus peak, times were taken from stimulus offset. Due to the high variability across participants, we plotted values extracted from the mean across participants and layers. (B) BOLD (n = 11) signal changes (%) of the initial peak (left), trough (middle), and post-stimulus peak (right) of the triphasic response for distance bins ≥4–6 mm. For BOLD TTPs and signal changes in individual layer compartments, see Supplementary Figure S13. For the corresponding VASO results across layers, see Supplementary Figure S12B and C.

The BOLD TTPs are shown in Figure 6A. Overall, TTPs are consistently increasing or decreasing across distance bins for all three phases of the response. For the BOLD initial peak, we found longest TTPs at closer distances and decreasing TTPs with distance. For the BOLD trough, we found longest TTPs at closer distances and decreasing TTPs with distance. For the BOLD post-stimulus we found shortest TTPs at closer distances (6–8 mm) and increasing TTPs with distance. Finally, we found only minor differences in TTPs across layer compartments which will not be interpreted further (see Supplementary Fig. S13A).

The respective BOLD signal changes at the corresponding TTPs are shown in Figure 6B. Also here, signal changes are overall consistently increasing or decreasing across distance bins for the initial peak and the trough of the triphasic response. For the BOLD initial peak, we found highest signal changes at closer distances and decreasing peak strengths for increasing distance. For the BOLD trough, we found increasing magnitudes (more negative signal changes) with distance from the peak activation. For the BOLD post-stimulus peak, the signal changes increase from the 6–8 mm to the 8–10 mm distance bins and then plateaus with minimal changes. Interestingly, the BOLD TTP and signal changes are inversely related for the trough (i.e., more negative signal changes for further distances occur at shorter TTPs) but not for the initial peak and post-stimulus peak. Finally, and unsurprisingly, we found increasing BOLD signal changes from deep to superficial layers in all phases and distance bins, which will not be interpreted further (see Supplementary Fig. S13B).

The triphasic response is not restricted to individual participants, the digit ROIs, stimulation of an individual digit, or BOLD data

The BOLD signal changes as a function of distance from the activation peak of individual participants are shown in Supplementary Figures S9 and S10. Qualitatively, we observed a pattern that resembles the group results in 5 out of 11 participants. Crucially, this seems to be related to the number of repetitions of stimulation we acquired for each participant. In five out of the seven participants for which we acquired >8 repetitions per digit, we observed a similar pattern as the group results. For four of the remaining six participants, we only acquired four repetitions per digit and the data quality may not be sufficient to detect these signal changes with respect to the noise in the data.

To make sure that the triphasic response we observed is not dependent on the signal fluctuations in the ROIs but changes as a function of distance from the peak voxel, we performed a control analysis in which we excluded data from the digit ROIs (for the new distance ROIs excluding the digit ROIs, see Supplementary Fig. S11A). The results are shown in Supplementary Figure S11B. Crucially, the triphasic response in more distant bins remains intact even if the digit ROIs are excluded.

To investigate whether the triphasic response is driven by the stimulation of a specific digit, we plotted the BOLD responses of one participant with high data quality (sub-16) for individual digits separately (Supplementary Fig. S11C). Note that this reduces the data going into the analysis by a factor of 3. Nevertheless, indications of a triphasic response are preserved in all individual digits in this participant. Even though this is not evidence that all digits contribute equally, this result shows that all digits seem to contribute to at least some degree.

We further investigated the VASO results for the five participants in which we could identify the triphasic response for more distant bins in the BOLD data (i.e., participants marked with a green box in Supplementary Figs. S9 and S10). The group-level VASO results (n = 5) are shown in Figure 5C. On the level of this sub-group (n = 5), a similar triphasic response can be observed in the VASO compared with the BOLD data. However, it is less clear due to higher noise levels and variability across participants in the VASO data (for VASO data from individual participants, see Supplementary Figs. S14 and S15). For completeness, the VASO TTPs and corresponding signal changes across layers are shown in Supplementary Figure S12.

## 6 Discussion

### 6.1 Summary of results

In the present study, we explored the laminar and spatio-temporal BOLD and CBV response characteristics to passive vibrotactile stimulation of three digit tips (30 seconds duration) in the human primary somatosensory cortex. To do so, we developed a new VASO sequence protocol with which we could image the entire depth of the central sulcus (coverage in slice direction: 28.38 mm) at high in-lane resolution (0.75 mm), relatively low slice thickness (1.29 mm), and acceptable nominal temporal resolution (3850 ms). With this protocol, we acquired depth-resolved BOLD and CBV data using VASO at 7T (f)MRI. Our goals were to test whether our newly developed sequence protocol would provide sufficient SNR to capture laminar BOLD and VASO responses to digit stimulation in contralateral human BA3b and to characterize VASO and BOLD signal changes across space and time, as far as SNR allows.

For BOLD, we found reliable digit representations in putative BA3b for all participants. For VASO, we found identifiable representations for all 3 digits in 7 out of 11 participants, and for 2 out of 3 digits in 2 further participants. Crucially, in some participants, this digit mapping using VASO was possible with limited data of as few as four repetitions per digit. Furthermore, we found that the responses to bottom-up digit stimulation across cortical depth followed the expected pattern of highest activation between middle and superficial cortical layers for VASO and increasing activation toward the cortical surface for BOLD. However, we found slightly negative responses for non-preferred digits, where the effect was largest (i.e., most negative) in superficial layers for both BOLD and VASO. In an exploratory analysis, we observed a triphasic response to the stimulation of non-preferred digits in the digit ROIs of the contralateral BA3b. Specifically, we found a small initial positive activation in response to stimulation of non-preferred digits, followed by a more sustained negative signal change compared with baseline and a positive post-stimulation response. This pattern was most prominent in the BOLD and to a certain extent also visible in the VASO data. Finally, we explored the triphasic pattern as a function of distance from each digit’s peak activation. We found that the initial peak decreased with distance from the activation hotspot of each digit, and the following deactivation increased with distance from the activation hotspot of each digit.

### 6.2 Mapping of digit representations using BOLD and VASO

One of the goals of the present study was to investigate to what extent our newly developed sequence protocol would be suitable for obtaining maps of digit representations in putative BA3b (i.e., the first area to receive detailed sensory input from individual digits; Penfield & Boldrey, 1937). Compared with previous investigations of laminar functional responses in the somatosensory domain (Yang et al., 2019; Yu et al., 2019, 2021), we increased the coverage in slice direction to 28.38 mm (compared with 18 mm), while reducing the slice thickness to 1.29 mm (compared with 1.8 mm) and keeping a relatively high in-plane resolution of 0.75 mm (compared with 0.71 mm) at the cost of nominal temporal resolution (3850 ms compared with 3490 ms). These changes enabled us to investigate CBV and BOLD responses to digit stimulation across the entire cortical landscape of putative BA3b at relatively high spatiotemporal resolution, which is crucial for flexible mapping of laminar digit representations on the postcentral gyrus (for an implementation on a Phillips platform with 13 slices at 0.79 × 0.79 × 1.5 mm resolution and a pairTR of 3000 ms, see de Oliveira et al., 2023).

With the new acquisition protocol, we found reliable digit representations based on the BOLD data in all participants (Figs. 2 and 3; Supplementary Figs. S3–S5) and the overall organization is in agreement with previous studies (Nelson & Chen, 2008; Sanchez-Panchuelo et al., 2010, 2012; Schweizer et al., 2008; Steinbach et al., 2024; Stringer et al., 2011). For the VASO data, we found weaker activation compared with BOLD, as expected due to the challenging SNR (Huber, 2014). Nevertheless, we were able to show activation clusters that matched the location of BOLD activity for all digits in 7 out of 11 participants (Fig. 2; Supplementary Figs. S3–S5). For two additional participants, we found clusters for two out of three digits that matched the BOLD activation. For 2 out of 11 participants, we found identifiable VASO responses for one digit only. These results seem comparable with those in the only other published study showing VASO responses in multiple individual digit regions of the human somatosensory cortex (de Oliveira et al., 2023). Specifically, de Oliveira et al. (2023) showed VASO maps that follow the expected organization in four out of six participants (∼66 %), which is similar to the ∼64 % reported here. Future studies will show whether this is due to individual participants’ VASO response characteristics in the somatosensory domain and/or amount of data acquired per participant.

In this context, we would like to highlight that the amount of data used in the present experiment is exceptionally small and the VASO results should be interpreted with caution. While we acquired only between 4 and 12 repetitions per digit across participants, for example, Huber et al. (2021) used 20 blocks of stimulation (30 s, each) with a similar setup. This is almost twice as much as for the participants who underwent the most scanning in our experiment. Furthermore, de Oliveira et al. (2023) acquired a similar amount of data for all participants, as we did for the participants with the most repetitions (approximately 12 repetitions of 30 s per digit). However, de Oliveira et al. (2023) used an active finger flexion task, while we used passive vibrotactile stimulation. Previous studies have shown that the location of digit representations resulting from active and passive tasks is similar, with activation strength being higher for active tasks (Sanders et al., 2023). Nevertheless, we believe that using passive vibrotactile stimulation is crucial for more applied experimental settings because it allows consistently accurate, experimenter-controlled administration of stimuli, compared with more variable active tasks or manual brushing. Finally, despite the small amount of data, a key benefit of the VASO sequence is that both BOLD- and CBV-weighted images are acquired in an interleaved fashion. As a result, the high sensitivity of BOLD can be used to guide the definition of ROIs, to then extract additional depth-resolved CBV responses with higher specificity.

With the restrictions of the limited VASO data in mind, we still extend the literature by providing insights into laminar spatial and temporal dynamics in response to passive stimulation of individual digits in humans. When investigating responses to stimulation of the preferred digit within the digit ROIs across cortical depth, unsurprisingly, we found the BOLD response to increase toward the cortical surface due to the well-known draining-vein effect (Fig. 3B; De Martino et al., 2013; Polimeni et al., 2010; Turner, 2002). In contrast, the VASO response showed a peak between middle and superficial cortical depths (Fig. 3B), as expected due to the higher specificity toward underlying neuronal populations of VASO. Here, the activation closer to middle cortical layers likely reflects feedforward thalamic input arriving in cortical layer 4. At first glance, the VASO peak response is located surprisingly close to the cortical surface compared with an expected response centered in gray matter as reported in VASO studies in the visual domain (e.g., Akbari et al., 2022; Dresbach et al., 2023). In part, this may be explained by the low cortical thickness of the primary somatosensory cortex (Fischl & Dale, 2000). Furthermore, because we found clear activation for all digits of all participants in BOLD but not VASO, we based our ROIs on the BOLD activation, which may include a larger number of surface vessels that will bias the VASO response toward CSF. Finally, we did not employ an explicit attention task in the present study. Therefore, it is conceivable that participants actively directed their attention toward the stimulation, which has been found to increase responses in superficial layers (e.g., Gau et al., 2020; Lawrence et al., 2019). Future studies in which the participants’ attention is controlled may investigate the laminar CBV response peak.

### 6.3 Negative responses in non-preferred digit ROIs

In our ROI analysis, we found slightly negative BOLD and VASO z-scores in response to stimulation of non-preferred digits for all ROIs (Fig. 3B). Negative BOLD responses (in terms of, e.g., GLM parameter estimates) in digit ROIs during contralateral stimulation of non-preferred digits have been observed in many studies using fMRI in humans (e.g., Khalife et al., 2022; Martuzzi et al., 2014, 2015; Van Der Zwaag et al., 2015). However, there are also studies that do not find these negative BOLD signal changes in ROIs of non-preferred digits (e.g., Ann Stringer et al., 2014; Kolasinski et al., 2016), while other authors show mixed results (e.g., Besle et al., 2014; Sanchez Panchuelo et al., 2018). Specifically, Sanchez Panchuelo et al. (2018) find negative BOLD responses for stimulation of non-preferred digits in some digit ROIs but not others (see their Fig. 4), whereas Besle et al. (2014) find negative BOLD responses for stimulation of non-preferred digits in the anterior portions of their digit ROIs (probably corresponding to BA3b). Furthermore, most studies that show negative BOLD responses in ROIs corresponding to non-preferred digits still report positive BOLD responses to stimulation of neighboring digits (e.g., Besle et al., 2014; Martuzzi et al., 2014). However, we found negative responses to BOLD and VASO already for neighboring digits in all our ROIs. This may be due to our ROI definition criteria (digit > other digits vs. digit > baseline) or due to the type of stimulation we employed. For example, Van Der Zwaag et al. (2015) compared manual with mechanical stimulation and found negative BOLD response in neighboring digit ROIs only for mechanical but not for manual stimulation. Our results are consistent with the investigation by Van Der Zwaag et al. (2015) given that we used piezoelectric stimulation. Finally, to our knowledge, the present report is the first paper showing that comparable negative VASO responses can be seen (for an OHBM abstract showing similar results, see Yang et al., 2023). Taken together, negative BOLD (and VASO) responses to stimulation of non-preferred digits are well established (although not reported unanimously) and our results are in line with the majority of the literature (for a model that implicates negative surround responses in the effects of aging, see Pleger et al., 2016).

### 6.4 The temporal evolution of the triphasic response

A much less studied aspect in the human primary somatosensory cortex is the temporal evolution of the response to contralateral stimulation of the digits. However, in an exploratory analysis, we found that during stimulation of non-preferred digits, our digit ROIs (especially ROIs corresponding to D2 and D4) showed a triphasic response, consisting of a small initial positive response, followed by a sustained signal decrease and a positive response after stimulus cessation (for similar results in the visual domain, see, e.g., Huber et al., 2014; Shmuel et al., 2006). Note that the term “triphasic response” has been used for similar signal fluctuations in the animal literature by, for example, Zhu and Connors (1999), Kleinfeld and Delaney (1996), and Derdikman et al. (2003). Furthermore, we found that the evolution of the triphasic response changes as a function of distance from the peak activation in response to stimulation of digits. Specifically, the magnitude of the initial peak decreased with distance from the activation center, whereas the magnitude of the trough and the post-stimulus peak increased with distance from the activation center. Other groups have also reported similar findings in regions of the somatosensory cortex representing the rat forepaw (e.g., Devor et al., 2005, 2007), rat whisker (Boorman et al., 2010), and squirrel monkey digits (e.g., Simons et al., 2007) using various invasive methods (e.g., laminar electrode array recordings, voltage-sensitive dye imaging, or two photon microscopy).

In humans, the temporal evolution of responses to digit stimulation in contralateral human BA3b is described in a much smaller number of studies and often with short stimulation periods. For example, the studies by Besle et al. (2014) and Khalife et al. (2022) showed small deflections below baseline in ROIs upon short stimulation of non-preferred digits after 1 second of vibrotactile stimulation. However, the full evolution of the response as we see it, with an initial positive response, a prolonged negative deflection during stimulation and another positive post-stimulus response are not visible in their studies. Potentially, longer stimulation times may be necessary for the full triphasic response to develop similar to the results reported in the present study and, for example, by Boorman et al. (2010) in rats. Therefore, future studies could vary the stimulus durations and investigate the evolution of the different phases of the triphasic response as a function of stimulus duration.

One study that investigated the temporal evolution of responses to digit stimulation in contralateral human BA3b to longer stimulus durations was carried out by Ann Stringer et al. (2014). The authors used air puff stimulation (2 Hz) to the distal finger pad with a 5 mm aperture and a 24-second on/off block design. However, they did not observe negative signal changes in non-preferred digit ROIs. Additionally, they performed a distance-based analysis similar to ours, in which they plotted normalized beta values as a function of radial distance from peak voxels of each digit in BA3b and BA1 separately. Interestingly, they did not observe negative beta values at any distance from the peak voxel, even at the maximum distance plotted (8 mm). In our data, we observed slight negative deflections below baseline already at 4–6 mm distance from the peak voxels and clearly at 6–8 mm (see Figs. 5 and 6). The discrepancy between our results and the findings by Ann Stringer et al. (2014) may be explained by several key differences in the setup or the analysis of the two studies. For example, Ann Stringer et al. (2014) used air puffs at 2 Hz as means of stimulation, while we used vibrotactile stimuli at 24 Hz. Given that the frequency of stimulation influences the neuronal and BOLD response magnitude or responding neuronal population in the somatosensory system, it is conceivable that the negative responses surrounding the positive response are similarly affected (Ngai et al., 1999; Sanganahalli et al., 2008). Furthermore, Ann Stringer et al. (2014) acquired fMRI data at a nominal resolution of 1 × 1 × 2 mm^3^, while we acquired data at a might higher resolution (0.75 × 0.75 × 1.29 mm^3^) which may lead to more partial voluming in their data compared with ours. Next, the authors defined the distances from a peak voxel radially (without specifying that a cortical mask was used), while we defined distances only within cortical gray matter. Finally, Ann Stringer et al. (2014) plotted beta values as a function of distance, which depend on an expected hemodynamic shape, whereas we used a model-independent approach and investigated mean signal changes [%]. Importantly, the triphasic response does not follow the evolution of the canonical hemodynamic response that is used to model fMRI data in GLM contexts (and hence estimate beta values). Therefore, a GLM might be at a disadvantage when examining negative responses compared with model-free approaches.

Finally, responses very similar to those reported in the present study have been observed after stimulation of the *ipsilateral* digits or median nerve using BOLD (e.g., Hlushchuk & Hari, 2006; Kastrup et al., 2008; Klingner et al., 2010, 2011), and/or cerebral blood flow (CBF) measurements (Mullinger et al., 2014; Schäfer et al., 2012). In this context, Boorman et al. (2010) investigated responses in regions surrounding the barrel of a stimulated whisker in the contralateral, as well as responses in the ipsilateral barrel cortex of rats using BOLD fMRI. Crucially, they found very similar responses in both cortices, which might indicate that similar mechanisms underlie the triphasic response in the ipsilateral (as reported by, e.g., Hlushchuk & Hari, 2006; Kastrup et al., 2008; Klingner et al., 2010, 2011) and the contralateral somatosensory system (as reported by us).

One aspect that has received very little attention in the literature so far is the timing and amplitude of the individual phases of the triphasic response (or similar responses). Therefore, we plotted TTPs and magnitude of BOLD signal changes for each of the three phases at each distance bin separately (Fig. 6; for laminar BOLD and VASO results, see Supplementary Figs. S13 and S12), which may help to shed light on the origin of the triphasic response. For example, it is noteworthy that the TTPs for the initial peak and the trough decrease with distance from the peak voxel of the stimulated digit, whereas for the post-stimulus peak, the TTP increases with distance from the peak voxel of the stimulated digit. This may hint toward different vascular or neuronal mechanisms underlying the different phases of the triphasic response. For example, it is conceivable that oxygen-rich blood being transported toward the region representing the stimulated digit leads to a traveling wave of increasingly large BOLD responses from more distant to closer regions (i.e., shorter TTPs and lower signal changes at larger distances and increasingly longer TTPs and higher signal changes smaller distances), giving rise to the initial peak. Similarly, the reverse could be true for the post-stimulus peak. Future studies with dedicated high-resolution vascular imaging (e.g., time-of-flight (Bollmann et al., 2022), multi-echo gradient recalled echo (Dresbach et al., 2024; Gulban et al., 2022) or 3D multi-echo epi (Gulban et al., 2025)), could help to delineate arterial and venous blood vessels to investigate the hemodynamic responses that give rise to the different phases of the triphasic response over time and space. Furthermore, higher quality laminar CBV data might help interpret the feedforward or feedback nature of the different phases of the triphasic response given that these processes are thought to target layer compartments differently and CBV responses are thought to be more specific to the underlying neuronal populations (Dumoulin et al., 2018; Huber et al., 2015; Lu et al., 2003).

### 6.5 Limitations and future directions

There are several limitations that have implications for the results discussed in the present manuscript.

Most importantly, the data presented were collected in the context of a larger study. Therefore, we were only able to acquire a limited amount of stimulation data for some participants (sometimes only four stimulation periods of 30 s per digit). This is especially detrimental for the VASO data, given its challenging SNR and low temporal efficiency. Specifically, other studies with a similar setup (e.g., Huber et al., 2021) acquired almost twice as much data (20 stimulation periods of 30 s) compared with the participants for whom we acquired the most data (12 stimulation periods of 30 s per digit). Nonetheless, we were able to obtain reasonable responses to digit stimulation in most participants, even in the VASO data. However, there is considerable variability in the data across participants regarding the triphasic response. Therefore, these exploratory results with respect to the timings and magnitudes of the phases of the triphasic response across distance bins have to be interpreted with caution. Especially given that we extracted TTPs and signal changes from the mean across participants, statistical testing would not be appropriate. Therefore, future studies with higher data quality on the single participant level will have to validate the present results.

Furthermore, due to the relatively low quality of the VASO data in some participants, we defined our regions of interest based on the consistently higher quality BOLD data. As a result, the ROIs may have been biased toward areas containing larger draining veins within the cortex. However, we believe that this is unlikely to be an issue at the cortical surface for multiple reasons. Most importantly, we defined the ROIs using the contrast of a single digit > other digits. The large pial veins will drain blood from multiple digit representations and, therefore, they are less likely to show up in the contrast images. Secondly, we restricted our analysis to voxels within gray matter based on an accurate manual segmentation. While this does not preclude signal from large vessels to leak into gray matter due to partial voluming in the coarser functional resolution, together with the fact that we used contrast images to define ROIs, we believe that this effect is negligible. Nevertheless, future studies could use higher quality VASO data to define the ROIs based on CBV measurements that are less biased toward the large draining veins.

Furthermore, the inter-trial period of our design did not allow a complete return to baseline for the BOLD and VASO responses. We used a 30 s on/off block design, which is used in many layer-fMRI studies, especially when also recording non-BOLD responses, because of a good tradeoff between detection sensitivity, temporal efficiency, and signal integrity (e.g., Dresbach et al., 2023; Faes et al., 2023; Huber et al., 2017; Pizzuti et al., 2023). Given that the triphasic response observed here is clearly locked to the onset of the stimulus, we do not believe that the effect is driven by preceding stimuli. However, because of partially overlapping responses, the exact quantification of the response timings and magnitudes in the event-related averages is difficult. In addition, the baseline calculation in the event-related averages may have slightly overestimated the baseline value. Therefore, the signal changes of the peaks may be slightly underestimated, while the signal changes of the troughs may be slightly overestimated. In future studies, a longer inter-stimulus period could be used to shed further light on the timing and magnitudes of the observed responses.

Another limitation of this study is that we cannot completely rule out the effect of prediction in the observed responses. That is because within each run, the order in which digits were stimulated across blocks showed limited variation (note, however, that only one digit was stimulated per block; for a complete description of which stimulation pattern[s] were used for which participant, see Supplementary Fig. S1 and Supplementary Table S1). Nevertheless, we argue that the effect of prediction in our results is negligible for several reasons. First, only a small proportion of stimulation blocks were likely influenced by prediction effects. Because the stimulation patterns were structured such that repetitions became recognizable only after at least two full cycles, early blocks were effectively unpredictable. Furthermore, stimulation patterns were alternated across runs, further minimizing expectancy. Even in the few cases where patterns repeated across runs, recognition would have required retention of a sequence presented over 20 minutes earlier, making strong prediction unlikely. When accounting for these factors across all participants and runs, a maximum of 33 out of 88 blocks could have been predictable, representing a limited fraction of the total dataset. Second, in block designs like in the present study, potential prediction effects would only influence the onset of stimulation, as participants immediately know which digit is being stimulated for the remainder of the block once the block begins. Taken together, while prediction effects across stimulation blocks cannot be ruled out completely in the present study due to the chosen stimulation design, the effect is likely to be small and comparable with many other layer-fMRI studies that used individual or a limited set of stimuli presented at fixed intervals (e.g., Budde et al., 2014; Chai et al., 2024; Han et al., 2025; Huber et al., 2015; Koopmans et al., 2010; Nothnagel et al., 2025; Shao et al., 2024; Vizioli et al., 2024). Nevertheless, to remove any residual effect of prediction on depth-dependent layer profiles in response to finger stimulation, future studies should use completely randomized stimulation orders across blocks.

## 7 Conclusion

We developed a sequence protocol with which we characterized hemodynamic responses to stimulation of individual digit tips across cortical depth at 0.75 mm in plane spatial resolution using BOLD and VASO fMRI. We could identify digit-specific ROIs in putative Brodmann area 3b, following the known anatomical organization. In the ROIs, the BOLD response increased toward the cortical surface due to the draining vein effect, while the VASO response was more shifted toward middle cortical layers, likely reflecting bottom-up input from the thalamus, as expected. Finally, we explored the temporal signal dynamics for BOLD and VASO as a function of distance from activation peaks resulting from stimulation of contralateral digits. With this analysis, we observed a triphasic response consisting of an initial peak and a subsequent negative deflection during stimulation, followed by a positive post-stimulus response in BOLD and to some extent in VASO. While similar responses were reported with invasive methods in animal models, to our knowledge, this is the first demonstration of such a triphasic response in the human contralateral somatosensory cortex during stimulation of individual digit tips. Furthermore, we investigated how the response features (timing and magnitude) of the three phases change as a function of distance from the activation center. Given that, unlike in animals, human experiments do not rely on anesthesia and can readily implement extensive behavioral testing, obtaining this effect in humans is an important step toward further uncovering the functional significance of the different aspects of the triphasic response.

## Supplementary Material

Supplementary Material

## Data Availability

Analysis code is available on GitHub: https://github.com/sdres/s1Anfunco. Raw data are available on OpenNeuro: https://openneuro.org/datasets/ds005238/versions/1.0.0.

## References

[IMAG.a.1157-b1] Abraham, A., Pedregosa, F., Eickenberg, M., Gervais, P., Mueller, A., Kossaifi, J., Gramfort, A., Thirion, B., & Varoquaux, G. (2014). Machine learning for neuroimaging with scikit-learn. Frontiers in Neuroinformatics, 8, 14. 10.3389/fninf.2014.00014 24600388 PMC3930868

[IMAG.a.1157-b2] Akbari, A., Bollmann, S., Ali, T. S., & Barth, M. (2022). Modelling the depth-dependent VASO and BOLD responses in human primary visual cortex. Human Brain Mapping, 44(2), 710–726. 10.1002/hbm.26094 36189837 PMC9842911

[IMAG.a.1157-b3] Ann Stringer, E., Qiao, P. G., Friedman, R. M., Holroyd, L., Newton, A. T., Gore, J. C., & Chen, L. M. (2014). Distinct fine-scale fMRI activation patterns of contra- and ipsilateral somatosensory areas 3b and 1 in humans. Human Brain Mapping, 35(9), 4841–4857. 10.1002/hbm.22517. 24692215 PMC4107165

[IMAG.a.1157-b5] Avants, B. B., Tustison, N. J., Song, G., Cook, P. A., Klein, A., & Gee, J. C. (2011). A reproducible evaluation of ANTs similarity metric performance in brain image registration. NeuroImage, 54(3), 2033–2044. 10.1016/j.neuroimage.2010.09.025 20851191 PMC3065962

[IMAG.a.1157-b6] Besle, J., Sánchez-Panchuelo, R.-M., Bowtell, R., Francis, S., & Schluppeck, D. (2013). Single-subject fMRI mapping at 7 T of the representation of fingertips in S1: A comparison of event-related and phase-encoding designs. Journal of Neurophysiology, 109(9), 2293–2305. 10.1152/jn.00499.2012 23427300 PMC3652218

[IMAG.a.1157-b7] Besle, J., Sánchez-Panchuelo, R.-M., Bowtell, R., Francis, S., & Schluppeck, D. (2014). Event-related fMRI at 7T reveals overlapping cortical representations for adjacent fingertips in S1 of individual subjects. Human Brain Mapping, 35(5), 2027–2043. 10.1002/hbm.22310 24014446 PMC4216413

[IMAG.a.1157-b8] Bollmann, S., Mattern, H., Bernier, M., Robinson, S. D., Park, D., Speck, O., & Polimeni, J. R. (2022). Imaging of the pial arterial vasculature of the human brain in vivo using high-resolution 7T time-of-flight angiography. eLife, 11, e71186. 10.7554/eLife.71186 35486089 PMC9150892

[IMAG.a.1157-b9] Boorman, L., Kennerley, A. J., Johnston, D., Jones, M., Zheng, Y., Redgrave, P., & Berwick, J. (2010). Negative blood oxygen level dependence in the rat: A model for investigating the role of suppression in neurovascular coupling. The Journal of Neuroscience, 30(12), 4285–4294. 10.1523/JNEUROSCI.6063-09.2010 20335464 PMC6634501

[IMAG.a.1157-b10] Budde, J., Shajan, G., Zaitsev, M., Scheffler, K., & Pohmann, R. (2014). Functional MRI in human subjects with gradient-echo and spin-echo EPI at 9.4 T. Magnetic Resonance in Medicine, 71(1), 209–218. 10.1002/mrm.24656 23447097

[IMAG.a.1157-b11] Chai, Y., Morgan, A. T., Handwerker, D. A., Li, L., Huber, L., Sutton, B. P., & Bandettini, P. A. (2024). Improving laminar fMRI specificity by reducing macrovascular bias revealed by respiration effects. Imaging Neuroscience, *2*, imag-2-00249. 10.1162/imag_a_00249 PMC12272271 40800537

[IMAG.a.1157-b12] Cox, R. W. (1996). AFNI: Software for analysis and visualization of functional magnetic resonance neuroimages. Computers and Biomedical Research, 29(3), 162–173. 10.1006/cbmr.1996.0014 8812068

[IMAG.a.1157-b13] De Martino, F., Zimmermann, J., Muckli, L., Ugurbil, K., Yacoub, E., & Goebel, R. (2013). Cortical depth dependent functional responses in humans at 7T: Improved specificity with 3D GRASE (N. Zhang, Ed.). PLoS One, 8(3), e60514. 10.1371/journal.pone.0060514 23533682 PMC3606277

[IMAG.a.1157-b14] de Oliveira, Í. A. F., Siero, J. C. W., Dumoulin, S. O., & van der Zwaag, W. (2023). Improved selectivity in 7 T digit mapping using VASO-CBV. Brain Topography, 36(1), 23–31. 10.1007/s10548-022-00932-x 36517699 PMC9834127

[IMAG.a.1157-b15] Derdikman, D., Hildesheim, R., Ahissar, E., Arieli, A., & Grinvald, A. (2003). Imaging spatiotemporal dynamics of surround inhibition in the barrels somatosensory cortex. The Journal of Neuroscience, 23(8), 3100–3105. 10.1523/JNEUROSCI.23-08-03100.2003 12716915 PMC6742298

[IMAG.a.1157-b16] Devor, A., Tian, P., Nishimura, N., Teng, I. C., Hillman, E. M. C., Narayanan, S. N., Ulbert, I., Boas, D. A., Kleinfeld, D., & Dale, A. M. (2007). Suppressed neuronal activity and concurrent arteriolar vasoconstriction may explain negative blood oxygenation level-dependent signal. The Journal of Neuroscience, 27(16), 4452–4459. 10.1523/JNEUROSCI.0134-07.2007 17442830 PMC2680207

[IMAG.a.1157-b17] Devor, A., Ulbert, I., Dunn, A. K., Narayanan, S. N., Jones, S. R., Andermann, M. L., Boas, D. A., & Dale, A. M. (2005). Coupling of the cortical hemodynamic response to cortical and thalamic neuronal activity. Proceedings of the National Academy of Sciences of the United States of America, 102(10), 3822–3827. 10.1073/pnas.0407789102 15734797 PMC550644

[IMAG.a.1157-b18] Dresbach, S., Huber, L., Gulban, O. F., & Goebel, R. (2023). Layer-fMRI VASO with short stimuli and event-related designs at 7 T. NeuroImage, 279, 120293. 10.1016/j.neuroimage.2023.120293 37562717

[IMAG.a.1157-b19] Dresbach, S., Huber, R., Gülban, Ö. F., Pizzuti, A., Trampel, R., Ivanov, D., Weiskopf, N., & Goebel, R. (2024). Characterisation of laminar and vascular spatiotemporal dynamics of CBV and BOLD signals using VASO and ME-GRE at 7T in humans. Imaging Neuroscience, 2, 1–16. 10.1162/imag_a_00263 PMC12290604 40800482

[IMAG.a.1157-b20] Dumoulin, S. O., Fracasso, A., van der Zwaag, W., Siero, J. C., & Petridou, N. (2018). Ultra-high field MRI: Advancing systems neuroscience towards mesoscopic human brain function. NeuroImage, 168(September 2016), 345–357. 10.1016/j.neuroimage.2017.01.028 28093360

[IMAG.a.1157-b21] Faes, L. K., De Martino, F., & Huber, L. (2023). Cerebral blood volume sensitive layer-fMRI in the human auditory cortex at 7T: Challenges and capabilities (J. Ahveninen, Ed.). PLoS One, 18(2), e0280855. 10.1371/journal.pone.0280855 36758009 PMC9910709

[IMAG.a.1157-b22] Fischl, B., & Dale, A. M. (2000). Measuring the thickness of the human cerebral cortex from magnetic resonance images. Proceedings of the National Academy of Sciences of the United States of America, 97(20), 11050–11055. 10.1073/pnas.200033797 10984517 PMC27146

[IMAG.a.1157-b23] Gau, R., Bazin, P.-L., Trampel, R., Turner, R., & Noppeney, U. (2020). Resolving multisensory and attentional influences across cortical depth in sensory cortices. eLife, 9, e46856. 10.7554/eLife.46856 31913119 PMC6984812

[IMAG.a.1157-b24] Guidi, M., Huber, L., Lampe, L., Gauthier, C. J., & Möller, H. E. (2016). Lamina-dependent calibrated BOLD response in human primary motor cortex. NeuroImage, 141, 250–261. 10.1016/j.neuroimage.2016.06.030 27364473

[IMAG.a.1157-b25] Gulban, O. F., Bollmann, S., Huber, L., Wagstyl, K., Goebel, R., Poser, B. A., Kay, K., & Ivanov, D. (2022). Mesoscopic in vivo human T 2 * dataset acquired using quantitative MRI at 7 Tesla. NeuroImage, 264, 119733. 10.1016/j.neuroimage.2022.119733 36375782

[IMAG.a.1157-b26] Gulban, O. F., Stirnberg, R., Tse, D., Pizzuti, A., Koiso, K., Archila-Melendez, M., Huber, L., Bollmann, S., Goebel, R., Kay, K., & Ivanov, D. (2025, March). In vivo reconstruction of Duvernoy’s postmortem vasculature images. bioRxiv, 10.1101/2025.03.23.644588

[IMAG.a.1157-b27] Han, S., Kim, D., Eun, S., Cho, H., & Kim, S.-G. (2025). 7T Spin-echo BOLD fMRI enhances spatial specificity in the human motor cortex during finger movement tasks. NeuroImage, 317, 121351. 10.1016/j.neuroimage.2025.121351 40581274

[IMAG.a.1157-b28] Harding-Forrester, S., & Feldman, D. E. (2018). Somatosensory maps. Handbook of Clinical Neurology, 151, 73–102. 10.1016/B978-0-444-63622-5.00004-8 29519481

[IMAG.a.1157-b29] Hlushchuk, Y., & Hari, R. (2006). Transient suppression of ipsilateral primary somatosensory cortex during tactile finger stimulation. Journal of Neuroscience, 26(21), 5819–5824. 10.1523/JNEUROSCI.5536-05.2006 16723540 PMC6675271

[IMAG.a.1157-b30] Hua, J., Jones, C. K., Qin, Q., & van Zijl, P. C. M. (2013). Implementation of vascular-space-occupancy MRI at 7T: 3D MT-VASO MRI at 7T. Magnetic Resonance in Medicine, 69(4), 1003–1013. 10.1002/mrm.24334 22585570 PMC4121129

[IMAG.a.1157-b31] Huber, L. (2014). Mapping human brain activity by functional magnetic resonance imaging of blood volume [Doctoral dissertation, University of Leipzig]. https://nbn-resolving.org/urn:nbn:de:bsz:15-qucosa-165252

[IMAG.a.1157-b32] Huber, L. (2020, June). Quality assurance measures for layer-fMRI time series: How to obtain them in LAYNII. Retrieved May 29, 2024, from https://layerfmri.com/2020/04/06/qa/

[IMAG.a.1157-b33] Huber, L., Finn, E. S., Handwerker, D. A., Bönstrup, M., Glen, D. R., Kashyap, S., Ivanov, D., Petridou, N., Marrett, S., Goense, J., Poser, B. A., & Bandettini, P. A. (2020). Sub-millimeter fMRI reveals multiple topographical digit representations that form action maps in human motor cortex. NeuroImage, 208(June 2019), 116463. 10.1016/j.neuroimage.2019.116463 31862526 PMC11829252

[IMAG.a.1157-b34] Huber, L., Goense, J., Kennerley, A. J., Ivanov, D., Krieger, S. N., Lepsien, J., Trampel, R., Turner, R., & Möller, H. E. (2014). Investigation of the neurovascular coupling in positive and negative BOLD responses in human brain at 7T. NeuroImage, 97, 349–362. 10.1016/j.neuroimage.2014.04.022 24742920

[IMAG.a.1157-b35] Huber, L., Goense, J., Kennerley, A. J., Trampel, R., Guidi, M., Reimer, E., Ivanov, D., Neef, N., Gauthier, C. J., Turner, R., & Möller, H. E. (2015). Cortical lamina-dependent blood volume changes in human brain at 7T. NeuroImage, 107, 23–33. 10.1016/j.neuroimage.2014.11.046 25479018

[IMAG.a.1157-b36] Huber, L., Handwerker, D. A., Jangraw, D. C., Chen, G., Hall, A., Stüber, C., Gonzalez-Castillo, J., Ivanov, D., Marrett, S., Guidi, M., Goense, J., Poser, B. A., & Bandettini, P. A. (2017). High-resolution CBV-fMRI allows mapping of laminar activity and connectivity of cortical input and output in human M1. Neuron, 96(6), 1253.e7–1263.e7. 10.1016/j.neuron.2017.11.005 29224727 PMC5739950

[IMAG.a.1157-b37] Huber, L., Kassavetis, P., Gulban, O. F., Hallett, M., & Horovitz, S. G. (2023). Laminar VASO fMRI in focal hand dystonia patients. Dystonia, 2, 10806. 10.3389/dyst.2023.10806 37035517 PMC10081516

[IMAG.a.1157-b38] Huber, L., Poser, B. A., Kaas, A. L., Fear, E. J., Dresbach, S., Berwick, J., Goebel, R., Turner, R., & Kennerley, A. J. (2021). Validating layer-specific VASO across species. NeuroImage, 237(May), 118195. 10.1016/j.neuroimage.2021.118195 34038769

[IMAG.a.1157-b39] Huber, L., Stirnberg, R., Morgan, A. T., Feinberg, D. A., Ehses, P., Knudsen, L., Gulban, O. F., Koiso, K., Gephart, I., Swegle, S., Wardle, S. G., Persichetti, A. S., Beckett, A. J. S., Stöcker, T., Boulant, N., Poser, B. A., & Bandettini, P. A. (2025). Short-term gradient imperfections in high-resolution EPI lead to Fuzzy Ripple artifacts. Magnetic Resonance in Medicine, 94(2), 571–587. 10.1002/mrm.30489 40173320 PMC12137764

[IMAG.a.1157-b40] Huber, L., Uludağ, K., & Möller, H. E. (2019). Non-BOLD contrast for laminar fMRI in humans: CBF, CBV, and CMRO2. NeuroImage, 197(February 2017), 742–760. 10.1016/j.neuroimage.2017.07.041 28736310 PMC12906290

[IMAG.a.1157-b41] Kalyani, A., Contier, O., Klemm, L., Azañon, E., Schreiber, S., Speck, O., Reichert, C., & Kuehn, E. (2023). Reduced dimension stimulus decoding and column-based modeling reveal architectural differences of primary somatosensory finger maps between younger and older adults. NeuroImage, 283, 120430. 10.1016/j.neuroimage.2023.120430 37923281

[IMAG.a.1157-b42] Kastrup, A., Baudewig, J., Schnaudigel, S., Huonker, R., Becker, L., Sohns, J. M., Dechent, P., Klingner, C., & Witte, O. W. (2008). Behavioral correlates of negative BOLD signal changes in the primary somatosensory cortex. NeuroImage, 41(4), 1364–1371. 10.1016/j.neuroimage.2008.03.049 18495495

[IMAG.a.1157-b43] Kazan, S. M., Huber, L., Flandin, G., Ivanov, D., Bandettini, P., & Weiskopf, N. (2017). Physiological basis of vascular autocalibration (VasA): Comparison to hypercapnia calibration methods. Magnetic Resonance in Medicine, 78(3), 1168–1173. 10.1002/mrm.26494 27851867 PMC5573956

[IMAG.a.1157-b44] Khalife, S., Francis, S. T., Schluppeck, D., Sánchez-Panchuelo, R.-M., & Besle, J. (2022). Fast event-related mapping of population fingertip tuning properties in human sensorimotor cortex at 7T. eneuro, 9(5), ENEURO.0069-22.2022. 10.1523/ENEURO.0069-22.2022 36194620 PMC9480917

[IMAG.a.1157-b45] Kleinfeld, D., & Delaney, K. (1996). Distributed representation of vibrissa movement in the upper layers of somatosensory cortex revealed with voltage-sensitive dyes. The Journal of Comparative Neurology, 375(1), 89–108. 10.1002/(SICI)1096-9861(19961104)375:1<89::AID-CNE6>3.0.CO;2-K 8913895

[IMAG.a.1157-b46] Klingner, C. M., Hasler, C., Brodoehl, S., & Witte, O. W. (2010). Dependence of the negative BOLD response on somatosensory stimulus intensity. NeuroImage, 53(1), 189–195. 10.1016/j.neuroimage.2010.05.087 20538064

[IMAG.a.1157-b47] Klingner, C. M., Huonker, R., Flemming, S., Hasler, C., Brodoehl, S., Preul, C., Burmeister, H., Kastrup, A., & Witte, O. W. (2011). Functional deactivations: Multiple ipsilateral brain areas engaged in the processing of somatosensory information. Human Brain Mapping, 32(1), 127–140. 10.1002/hbm.21006 21157879 PMC6870510

[IMAG.a.1157-b48] Kolasinski, X. J., Makin, T. R., Jbabdi, S., Clare, X. S., Stagg, C. J., & Johansen-berg, H. (2016). Investigating the stability of fine-grain digit somatotopy in individual human participants. Journal of Neuroscience, 36(4), 1113–1127. 10.1523/JNEUROSCI.1742-15.2016 26818501 PMC4728720

[IMAG.a.1157-b49] Koopmans, P. J., Barth, M., & Norris, D. G. (2010). Layer-specific BOLD activation in human V1. Human Brain Mapping, 31(9), 1297–1304. 10.1002/hbm.20936 20082333 PMC6870878

[IMAG.a.1157-b50] Kuehn, E., & Pleger, B. (2020). Encoding schemes in somatosensation: From micro- to meta-topography. NeuroImage, 223(August), 117255. 10.1016/j.neuroimage.2020.117255 32800990

[IMAG.a.1157-b51] Lawrence, S. J., Norris, D. G., & De Lange, F. P. (2019). Dissociable laminar profiles of concurrent bottom-up and top-down modulation in the human visual cortex. eLife, 8, e44422. 10.7554/eLife.44422 31063127 PMC6538372

[IMAG.a.1157-b52] Liu, P., Doehler, J., Henschke, J. U., Northall, A., Serian, A., Schwarzkopf, D. S., Speck, O., Pakan, J. M., & Kuehn, E. (2023, December). A layer-specific model of cortical sensory aging. Neuroscience. 10.1101/2023.12.01.567841

[IMAG.a.1157-b53] Lu, H., Golay, X., Pekar, J. J., & Van Zijl, P. C. (2003). Functional magnetic resonance imaging based on changes in vascular space occupancy. Magnetic Resonance in Medicine, 50(2), 263–274. 10.1002/mrm.10519 12876702

[IMAG.a.1157-b54] Marquardt, I., Schneider, M., Gulban, O. F., Ivanov, D., & Uludağ, K. (2018). Cortical depth profiles of luminance contrast responses in human V1 and V2 using 7 T fMRI. Human Brain Mapping, 39(7), 2812–2827. 10.1002/hbm.24042 29575494 PMC6866457

[IMAG.a.1157-b55] Marques, J. P., Kober, T., Krueger, G., van der Zwaag, W., Van de Moortele, P. F., & Gruetter, R. (2010). MP2RAGE, a self bias-field corrected sequence for improved segmentation and T1-mapping at high field. NeuroImage, 49(2), 1271–1281. 10.1016/j.neuroimage.2009.10.002 19819338

[IMAG.a.1157-b56] Marshall, W. E., Woolsey, C. N., & Bard, P. (1937). Cortical representation of tactile sensibility as indicated by cortical potentials. Science, 85, 388–390. 10.1126/science.85.2207.388 17777206

[IMAG.a.1157-b57] Martuzzi, R., Van Der Zwaag, W., Dieguez, S., Serino, A., Gruetter, R., & Blanke, O. (2015). Distinct contributions of Brodmann areas 1 and 2 to body ownership. Social Cognitive and Affective Neuroscience, 10(11), 1449–1459. 10.1093/scan/nsv031 25809404 PMC4631141

[IMAG.a.1157-b58] Martuzzi, R., van der Zwaag, W., Farthouat, J., Gruetter, R., & Blanke, O. (2014). Human finger somatotopy in areas 3b, 1, and 2: A 7T fMRI study using a natural stimulus. Human Brain Mapping, 35(1), 213–226. 10.1002/hbm.22172 22965769 PMC6869627

[IMAG.a.1157-b59] Merzenich, M. M., Kaas, J. H., Sur, M., & Lin, C.-S. (1978). Double representation of the body surface within cytoarchitectonic area 3b and 1 in “SI” in the owl monkey (aotus trivirgatus). Journal of Comparative Neurology, 181(1), 41–73. 10.1002/cne.901810104 98537

[IMAG.a.1157-b60] Merzenich, M. M., Nelson, R. J., Kaas, J. H., Stryker, M. P., Jenkins, W. M., Zook, J. M., Cynader, M. S., & Schoppmann, A. (1987). Variability in hand surface representations in areas 3b and 1 in adult owl and squirrel monkeys. Journal of Comparative Neurology, 258(2), 281–296. 10.1002/cne.902580208 3584541

[IMAG.a.1157-b61] Mullinger, K., Mayhew, S., Bagshaw, A., Bowtell, R., & Francis, S. (2014). Evidence that the negative BOLD response is neuronal in origin: A simultaneous EEG–BOLD–CBF study in humans. NeuroImage, 94, 263–274. 10.1016/j.neuroimage.2014.02.029 24632092

[IMAG.a.1157-b62] Nakamura, S., Nakaura, T., Kidoh, M., Awai, K., Namimoto, T., Yoshinaka, I., Harada, K., & Yamashita, Y. (2016). Efficacy of the projection onto convex sets (POCS) algorithm at Gd-EOB-DTPA-enhanced hepatobiliary-phase hepatic MRI. SpringerPlus, 5(1), 1311. 10.1186/s40064-016-2968-9 27547685 PMC4978650

[IMAG.a.1157-b63] Nelson, A. J., & Chen, R. (2008). Digit somatotopy within cortical areas of the postcentral gyrus in humans. Cerebral Cortex, 18(10), 2341–2351. 10.1093/cercor/bhm257 18245039

[IMAG.a.1157-b64] Ngai, A. C., Jolley, M. A., D’Ambrosio, R., Meno, J. R., & Winn, H. (1999). Frequency-dependent changes in cerebral blood flow and evoked potentials during somatosensory stimulation in the rat. Brain Research, 837(1–2), 221–228. 10.1016/S0006-8993(99)01649-2 10434006

[IMAG.a.1157-b65] Nothnagel, N., Morgan, A. T., Muckli, L., & Goense, J. (2025). Revealing layer-specific cortical activity in human M1 using high-resolution line-scanning fMRI. Imaging Neuroscience, 3, imag_a_00477. 10.1162/imag_a_00477 PMC12319926 40800885

[IMAG.a.1157-b66] Ogawa, S., Lee, T.-M., Kay, A. R., & Tank, D. W. (1990). Brain magnetic resonance imaging with contrast dependent on blood oxygenation. Proceedings of the National Academy of Sciences of the United States of America, 87(24), 9868–9872. 10.1073/pnas.87.24.9868 2124706 PMC55275

[IMAG.a.1157-b67] Olman, C. A., Inati, S., & Heeger, D. J. (2007). The effect of large veins on spatial localization with GE BOLD at 3 T: Displacement, not blurring. NeuroImage, 34(3), 1126–1135. 10.1016/j.neuroimage.2006.08.045 17157534

[IMAG.a.1157-b68] Penfield, W., & Boldrey, E. (1937). Somatic motor and sensory representation in the cerebral cortex of man as studied by electrical stimulation. Brain, 60(4), 389–443. 10.1093/brain/60.4.389

[IMAG.a.1157-b69] Pizzuti, A., Huber, L., Gulban, O. F., Benitez-Andonegui, A., Peters, J., & Goebel, R. (2023). Imaging the columnar functional organization of human area MT+ to axis-of-motion stimuli using VASO at 7 Tesla. Cerebral Cortex, 33(13), 8693–8711. 10.1093/cercor/bhad151 37254796 PMC10321107

[IMAG.a.1157-b70] Pleger, B., Wilimzig, C., Nicolas, V., Kalisch, T., Ragert, P., Tegenthoff, M., & Dinse, H. R. (2016). A complementary role of intracortical inhibition in age-related tactile degradation and its remodelling in humans. Scientific Reports, 6(1), 27388. 10.1038/srep27388 27302219 PMC4908433

[IMAG.a.1157-b71] Polimeni, J. R., Fischl, B., Greve, D. N., & Wald, L. L. (2010). Laminar analysis of 7T BOLD using an imposed spatial activation pattern in human V1. NeuroImage, 52(4), 1334–1346. 10.1016/j.neuroimage.2010.05.005 20460157 PMC3130346

[IMAG.a.1157-b72] Poser, B. A., Koopmans, P. J., Witzel, T., Wald, L. L., & Barth, M. (2010). Three dimensional echo-planar imaging at 7 Tesla. NeuroImage, 51(1), 261–266. 10.1016/j.neuroimage.2010.01.108 20139009 PMC2853246

[IMAG.a.1157-b73] Power, J. D., Mitra, A., Laumann, T. O., Snyder, A. Z., Schlaggar, B. L., & Petersen, S. E. (2014). Methods to detect, characterize, and remove motion artifact in resting state fMRI. NeuroImage, 84, 320–341. 10.1016/j.neuroimage.2013.08.048 23994314 PMC3849338

[IMAG.a.1157-b74] Puckett, A. M., Bollmann, S., Barth, M., & Cunnington, R. (2017). Measuring the effects of attention to individual fingertips in somatosensory cortex using ultra-high field (7T) fMRI. NeuroImage, 161, 179–187. 10.1016/j.neuroimage.2017.08.014 28801252

[IMAG.a.1157-b75] Puckett, A. M., Bollmann, S., Junday, K., Barth, M., & Cunnington, R. (2020). Bayesian population receptive field modeling in human somatosensory cortex. NeuroImage, 208, 116465. 10.1016/j.neuroimage.2019.116465 31863915

[IMAG.a.1157-b76] Sanchez-Panchuelo, R. M., Besle, J., Beckett, A., Bowtell, R., Schluppeck, D., & Francis, S. (2012). Within-digit functional parcellation of brodmann areas of the human primary somatosensory cortex using functional magnetic resonance imaging at 7 tesla. Journal of Neuroscience, 32(45), 15815–15822. 10.1523/JNEUROSCI.2501-12.2012 23136420 PMC6621625

[IMAG.a.1157-b77] Sánchez-Panchuelo, R. M., Besle, J., Mougin, O., Gowland, P., Bowtell, R., Schluppeck, D., & Francis, S. (2014). Regional structural differences across functionally parcellated Brodmann areas of human primary somatosensory cortex. NeuroImage, (93 Pt 2), 221–230. 10.1016/j.neuroimage.2013.03.044 23558101

[IMAG.a.1157-b78] Sanchez Panchuelo, R. M., Besle, J., Schluppeck, D., Humberstone, M., & Francis, S. (2018). Somatotopy in the human somatosensory system. Frontiers in Human Neuroscience, 12, 235. 10.3389/fnhum.2018.00235 29950980 PMC6008546

[IMAG.a.1157-b79] Sanchez-Panchuelo, R. M., Francis, S., Bowtell, R., & Schluppeck, D. (2010). Mapping human somatosensory cortex in individual subjects with 7T functional MRI. Journal of Neurophysiology, 103(5), 2544–2556. 10.1152/jn.01017.2009 20164393 PMC2867563

[IMAG.a.1157-b80] Sanders, Z.-B., Dempsey-Jones, H., Wesselink, D. B., Edmondson, L. R., Puckett, A. M., Saal, H. P., & Makin, T. R. (2023). Similar somatotopy for active and passive digit representation in primary somatosensory cortex. Human Brain Mapping, 44(9), 3568–3585. 10.1002/hbm.26298 37145934 PMC10203813

[IMAG.a.1157-b81] Sanganahalli, B. G., Herman, P., & Hyder, F. (2008). Frequency-dependent tactile responses in rat brain measured by functional MRI. NMR in Biomedicine, 21(4), 410–416. 10.1002/nbm.1259 18435491 PMC2774500

[IMAG.a.1157-b82] Schäfer, K., Blankenburg, F., Kupers, R., Grüner, J. M., Law, I., Lauritzen, M., & Larsson, H. B. (2012). Negative BOLD signal changes in ipsilateral primary somatosensory cortex are associated with perfusion decreases and behavioral evidence for functional inhibition. NeuroImage, 59(4), 3119–3127. 10.1016/j.neuroimage.2011.11.085 22155327

[IMAG.a.1157-b83] Schluppeck, D., Sanchez-Panchuelo, R. M., & Francis, S. T. (2018). Exploring structure and function of sensory cortex with 7 T MRI. NeuroImage, 164(January 2017), 10–17. 10.1016/j.neuroimage.2017.01.081 28161312

[IMAG.a.1157-b84] Schweisfurth, M. A., Frahm, J., & Schweizer, R. (2014). Individual fMRI maps of all phalanges and digit bases of all fingers in human primary somatosensory cortex. Frontiers in Human Neuroscience, 8(September), 1769–1785. 10.3389/fnhum.2014.00658 PMC4151507 25228867

[IMAG.a.1157-b85] Schweizer, R., Voit, D., & Frahm, J. (2008). Finger representations in human primary somatosensory cortex as revealed by high-resolution functional MRI of tactile stimulation. NeuroImage, 42(1), 28–35. 10.1016/j.neuroimage.2008.04.184 18550386

[IMAG.a.1157-b86] Shao, X., Guo, F., Kim, J., Ress, D., Zhao, C., Shou, Q., Jann, K., & Wang, D. J. (2024). Laminar multi-contrast fMRI at 7T allows differentiation of neuronal excitation and inhibition underlying positive and negative BOLD responses. Imaging Neuroscience, 2, imag-2-00311. 10.1162/imag_a_00311 PMC12254000 40656773

[IMAG.a.1157-b87] Shmuel, A., Augath, M., Oeltermann, A., & Logothetis, N. K. (2006). Negative functional MRI response correlates with decreases in neuronal activity in monkey visual area V1. Nature Neuroscience, 9(4), 569–577. 10.1038/nn1675 16547508

[IMAG.a.1157-b88] Simons, S. B., Chiu, J., Favorov, O. V., Whitsel, B. L., & Tommerdahl, M. (2007). Duration-dependent response of SI to vibrotactile stimulation in squirrel monkey. Journal of Neurophysiology, 97(3), 2121–2129. 10.1152/jn.00513.2006 17035362

[IMAG.a.1157-b89] Steinbach, T., Eck, J., Timmers, I., Biggs, E. E., Goebel, R., Schweizer, R., & Kaas, A. L. (2024). Tactile stimulation designs adapted to clinical settings result in reliable fMRI-based somatosensory digit maps. BMC Neuroscience, 25(1), 47. 10.1186/s12868-024-00892-x 39354349 PMC11443901

[IMAG.a.1157-b90] Steinbach, T., Eck, J., Zephyr, R., Goebel, R., Schweizer, R., & Kaas, A. (2023). Upper-limb immobilization changes fMRI-based somatosensory digit maps and tactile acuity. https://figshare.com/articles/conference_contribution/Steinbach_et_al_OHBM_2023/26135296

[IMAG.a.1157-b91] Stoll, S., Luesebrink, F., Schwarzkopf, D. S., Mattern, H., Liu, P., Noelle, J., & Kuehn, K. (2025). Modeling 2D spatio-tactile population receptive fields of the fingertip in human primary somatosensory cortex. bioRxiv. 10.1101/2025.05.24.655840 PMC13214571 42212224

[IMAG.a.1157-b92] Stringer, E. A., Chen, L. M., Friedman, R. M., Gatenby, C., & Gore, J. C. (2011). Differentiation of somatosensory cortices by high-resolution fMRI at 7T. NeuroImage, 54(2), 1012–1020. 10.1016/j.neuroimage.2010.09.058 20887793 PMC4270280

[IMAG.a.1157-b93] Talagala, S. L., Sarlls, J. E., Liu, S., & Inati, S. J. (2016). Improvement of temporal signal-to-noise ratio of GRAPPA accelerated echo planar imaging using a FLASH based calibration scan. Magnetic Resonance in Medicine, 75(6), 2362–2371. 10.1002/mrm.25846 26192822 PMC4720593

[IMAG.a.1157-b94] Turner, R. (2002). How much codex can a vein drain? Downstream dilution of activation-related cerebral blood oxygenation changes. NeuroImage, 16(4), 1062–1067. 10.1006/nimg.2002.1082 12202093

[IMAG.a.1157-b95] Van Der Zwaag, W., Gruetter, R., & Martuzzi, R. (2015). Stroking or buzzing? A comparison of somatosensory touch stimuli using 7 Tesla fMRI (A. J. Schwarz, Ed.). PLoS One, 10(8), e0134610. 10.1371/journal.pone.0134610 26285027 PMC4540472

[IMAG.a.1157-b96] Vizioli, L., Moeller, S., Dowdle, L., Grant, A., Sadeghi-Tarakameh, A., Eryaman, Y., Lagore R, R. L., Auerbach, E., Adriany, G., Knudsen, L., Martino, F. D., Faes, L., Yacoub, E., & Uğurbil, K. (2024, December). Spanning spatial scales with functional imaging in the human brain; initial experiences at 10.5 Tesla. bioRxiv. 10.1101/2024.12.20.629800

[IMAG.a.1157-b97] Woolrich, M. W., Ripley, B. D., Brady, M., & Smith, S. M. (2001). Temporal autocorrelation in univariate linear modeling of FMRI data. NeuroImage, 14(6), 1370–1386. 10.1006/nimg.2001.0931 11707093

[IMAG.a.1157-b98] Yang, J., Huber, L., Yu, Y., Chai, Y., Khojandi, A., & Bandettini, P. A. (2019). High-resolution fMRI maps of columnar organization in human primary somatosensory cortex. ISMRM, 27, 0617. https://archive.ismrm.org/2019/0617.html

[IMAG.a.1157-b99] Yang, J., Yu, Y., Huber, L., Fukunaga, M., Sadato, N., & Bandettini, P. (2023). Layer-specific finger representations in human area 3b. OHBM, Poster number 2617. Retrieved October 15, 2025, from https://www.youtube.com/watch?v=RBaKnDVdI18

[IMAG.a.1157-b100] Yu, Y., Huber, L., Yang, J., Fukunaga, M., Chai, Y., Jangraw, D. C., Chen, G., Handwerker, D. A., Molfese, P. J., Ejima, Y., Sadato, N., Wu, J., & Bandettini, P. A. (2021). Layer-specific activation in human primary somatosensory cortex during tactile temporal prediction error processing. NeuroImage, 248(December 2021), 118867. 10.1016/j.neuroimage.2021.118867 34974114 PMC11835052

[IMAG.a.1157-b101] Yu, Y., Huber, L., Yang, J., Jangraw, D. C., Handwerker, D. A., Molfese, P. J., Chen, G., Ejima, Y., Wu, J., & Bandettini, P. A. (2019). Layer-specific activation of sensory input and predictive feedback in the human primary somatosensory cortex. Science Advances, 5(5), 1–10. 10.1126/sciadv.aav9053 PMC6520017 31106273

[IMAG.a.1157-b102] Yushkevich, P. A., Piven, J., Hazlett, H. C., Smith, R. G., Ho, S., Gee, J. C., & Gerig, G. (2006). User-guided 3D active contour segmentation of anatomical structures: Significantly improved efficiency and reliability. NeuroImage, 31(3), 1116–1128. 10.1016/j.neuroimage.2006.01.015 16545965

[IMAG.a.1157-b103] Zhang, Y., Brady, M., & Smith, S. (2001). Segmentation of brain MR images through a hidden Markov random field model and the expectation-maximization algorithm. IEEE Transactions on Medical Imaging, 20(1), 45–57. 10.1109/42.906424 11293691

[IMAG.a.1157-b104] Zhu, J. J., & Connors, B. W. (1999). Intrinsic firing patterns and whisker-evoked synaptic responses of neurons in the rat barrel cortex. Journal of Neurophysiology, 81(3), 1171–1183. 10.1152/jn.1999.81.3.1171 10085344

