# Supplementary material for: Laminar CBV and BOLD response characteristics over time and space in the human primary somatosensory cortex at 7T
Source: Imaging Neurosci (Camb). 2026 Mar 27;4:IMAG.a.1157. doi: 10.1162/IMAG.a.1157 (PMC13034615; doi:10.1162/IMAG.a.1157)
Supplement: Supplementary Material [file IMAG.a.1157_supp.pdf]

Supplementary Materials

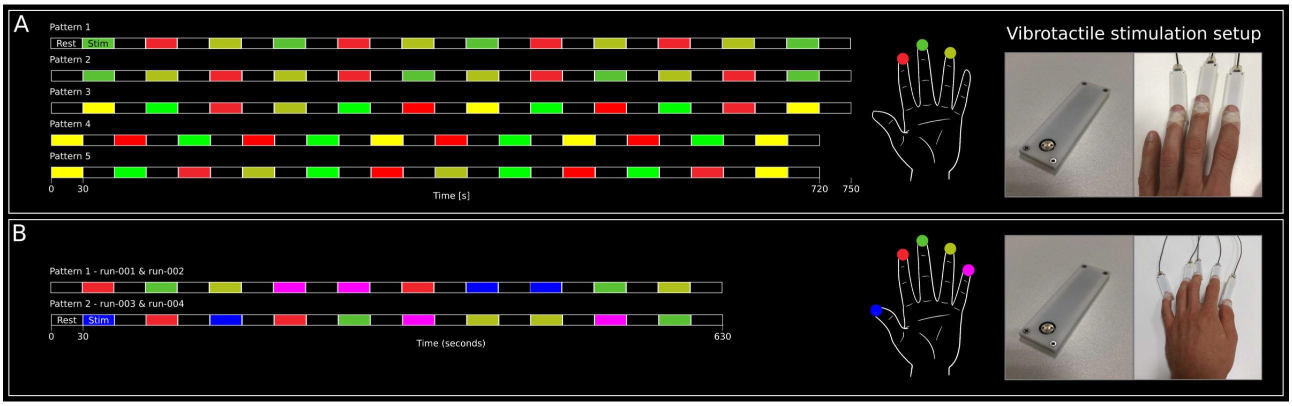

Figure S1: Stimulation pattern details. (**A**) Left: All stimulation patterns that were used in at least one participant. For a list which pattern(s) was (were) used in which participant, see Supplementary Table 1. Right: Color code of digits and photo of stimulator individually, as well as the stimulators attached to the digit-tips with medical tape. (**B**) Left: For participant sub-12, we stimulated the thumb (D1) and little finger (D5) of the left hand, in addition to D2-D4. Increasing the duration of each run to accommodate the stimulation of additional digits would have been detrimental for participant comfort. Therefore, we reduced the run duration by only including 2 repetitions/digit in each run (resulting in a run duration of 10.5 minutes, including 30 seconds of initial rest). To account for the reduction of repetitions per digit, we acquired 4 runs of stimulation for this participant, resulting in 8 repetitions/digit. Also here, we generated 2 stimulation patterns. For run-001 and run-002 we used pattern 1, while for run-003 and run-004 we used pattern 2. Right: Same as (**A**), but for all 5 digit-tips

Table 1: Session overview of participants.

Participant	Repetitions/digit	Digits stimulated	Stimulation pattern(s)
sub-01-sub-04	Pilots	-	-
sub-05	4	3	2
sub-06	4	3	5
sub-07	8	3	4, 3
sub-08	Session aborted due to participant discomfort	-	-
sub-09	4	3	1
sub-10	4	3	1
sub-11	Problems during scanning	-	-
sub-12	8	5	1, 1, 2, 2 (see Supplementary Figure S1B)
sub-13	Problems during scanning	-	-
sub-14	8	3	1, 2
sub-15	12	3	1, 2, 1
sub-16	12	3	1, 2, 1
sub-17	12	3	1, 2, 1
sub-18	12	3	1, 2, 1

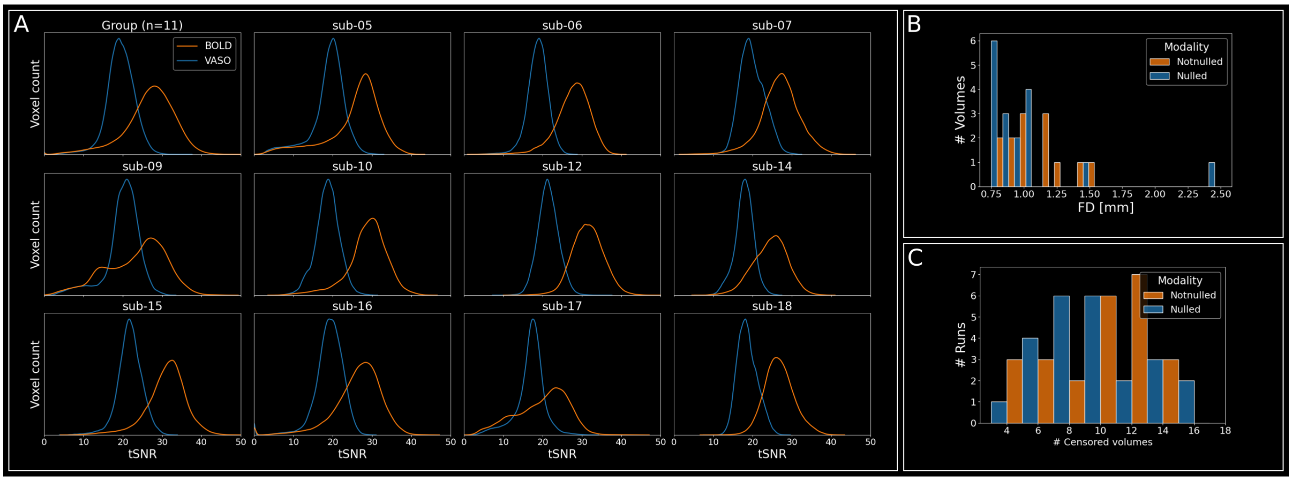

Figure S2: Quality assessment A TSNR values for BOLD and VASO on the group level (n = 11) and for each participant separately. Values were extracted from the "perimeter chunk" generated by the *LN2_MULTILATERATE* program (i.e. the geodesic disc propagated across cortical depth from in which digit specific ROIs were generated; see Figure 3A). **B** Histogram of the number of volumes showing framewise displacement (FD) > 0.75 mm (in-plane voxel resolution) of all participants and runs for nulled and notnulled data separately. A total of 9198 volumes (nulled and notnulled combined) was acquired across participants. Of those, 30 volumes (<0.33%) showed (FDs) greater than our voxel size (0.75 mm). 13 (in 6 individual runs) of those were in notnulled and 17 (in 9 individual runs) were in nulled time series. **C** Histogram of the number of runs with a certain number of censored volumes for nulled and notnulled timeseries separately as given by the *fsl_motion_outliers* program.

Table 2: Coordinates of central "control point" provided to the program *LN2_MULTILATERATE*. The program *LN2_MULTILATERATE* takes a minimum of 3 inputs. A tissue segmentation, a radius for the geodesic disc and a control points file. The control point(s) has (have) to be superimposed on the mid-gray-matter file which, in turn, is given by the program *LN2_LAYERS*. Specifically, the mid gray-matter demarcates the middle gray-matter voxel with a value of 1 while other voxels have a value of 0 and the control points file has to be identical to the mid-gray-matter file but with a voxel value of 2 instead of 1 at the desired center location of the geodesic disc. This table contains the x-, y-, and z-coordinates of the central point of each participant's geodesic disc. Central points and radius (12 mm) were chosen to encompass the main BOLD activation clusters of all contrasts maps (digit > other digits). If necessary, other control points can be specified to control the rotation of the geodesic disc. However, we did not choose this option.

Participant	x-coordinate	y-coordinate	z-coordinate
sub-05	281	209	120
sub-06	267	171	76
sub-07	275	206	100
sub-09	254	164	110
sub-10	335	174	105
sub-12	247	175	110
sub-14	274	170	100
sub-15	241	178	105
sub-16	259	190	135
sub-17	192	203	100
sub-18	258	159	100

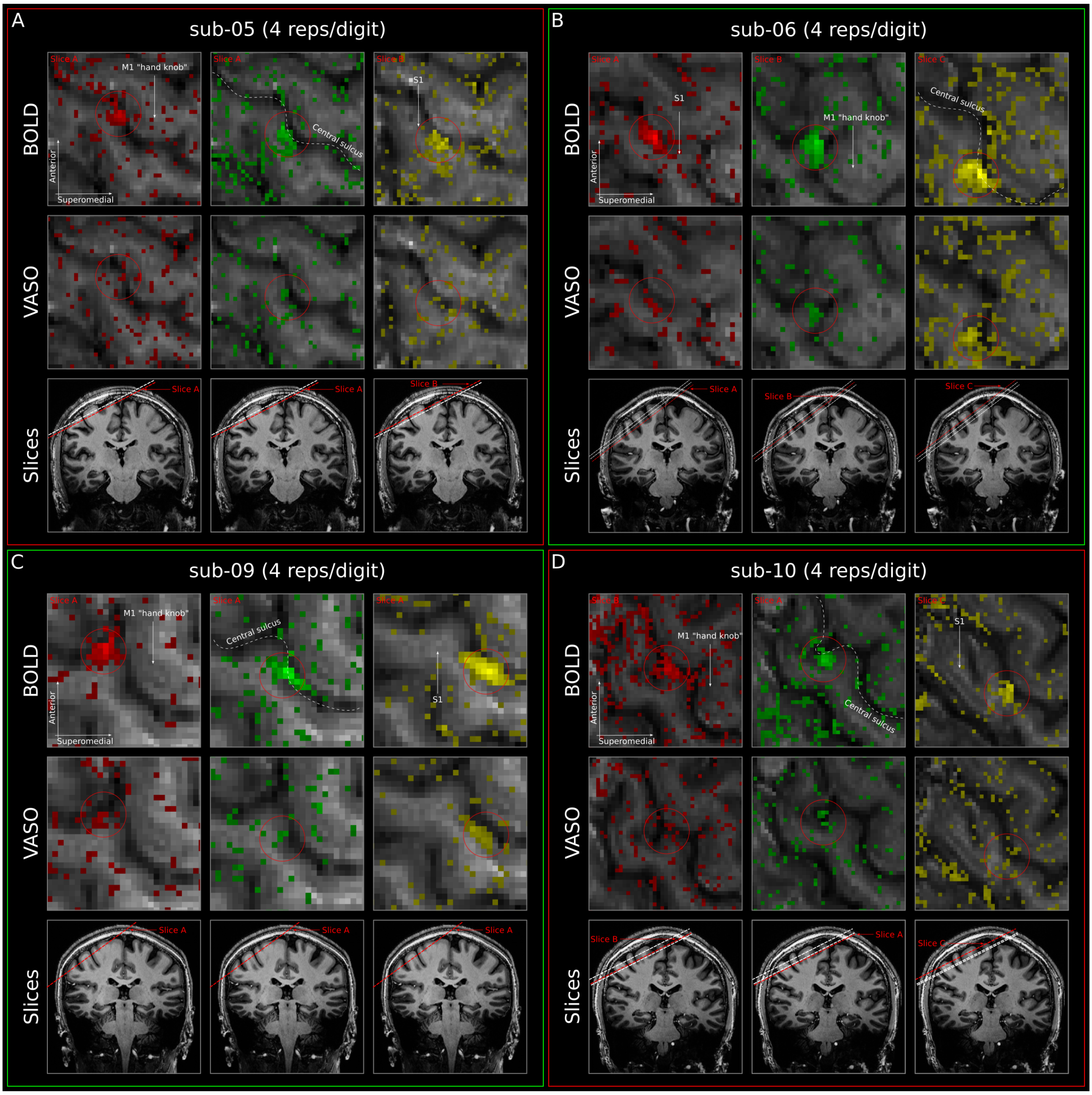

Figure S3: Stimulation results for participants sub-05-sub-10. Same as Figure 2B but for participants sub-05 (**A**), sub-06 (**B**), sub-09 (**C**), and sub-10 (**D**). A green box around the participant's plots indicates that we could identify 3/3 digit representations based on the VASO data, an orange box around the participant's plots indicates that we could identify 2/3 digit representations based on the VASO data, and a red box around the participant's plots indicates that we could identify only 1/3 digit representations based on the VASO data. Colorbars and identification of digit-clusters are the same as in Figure 2

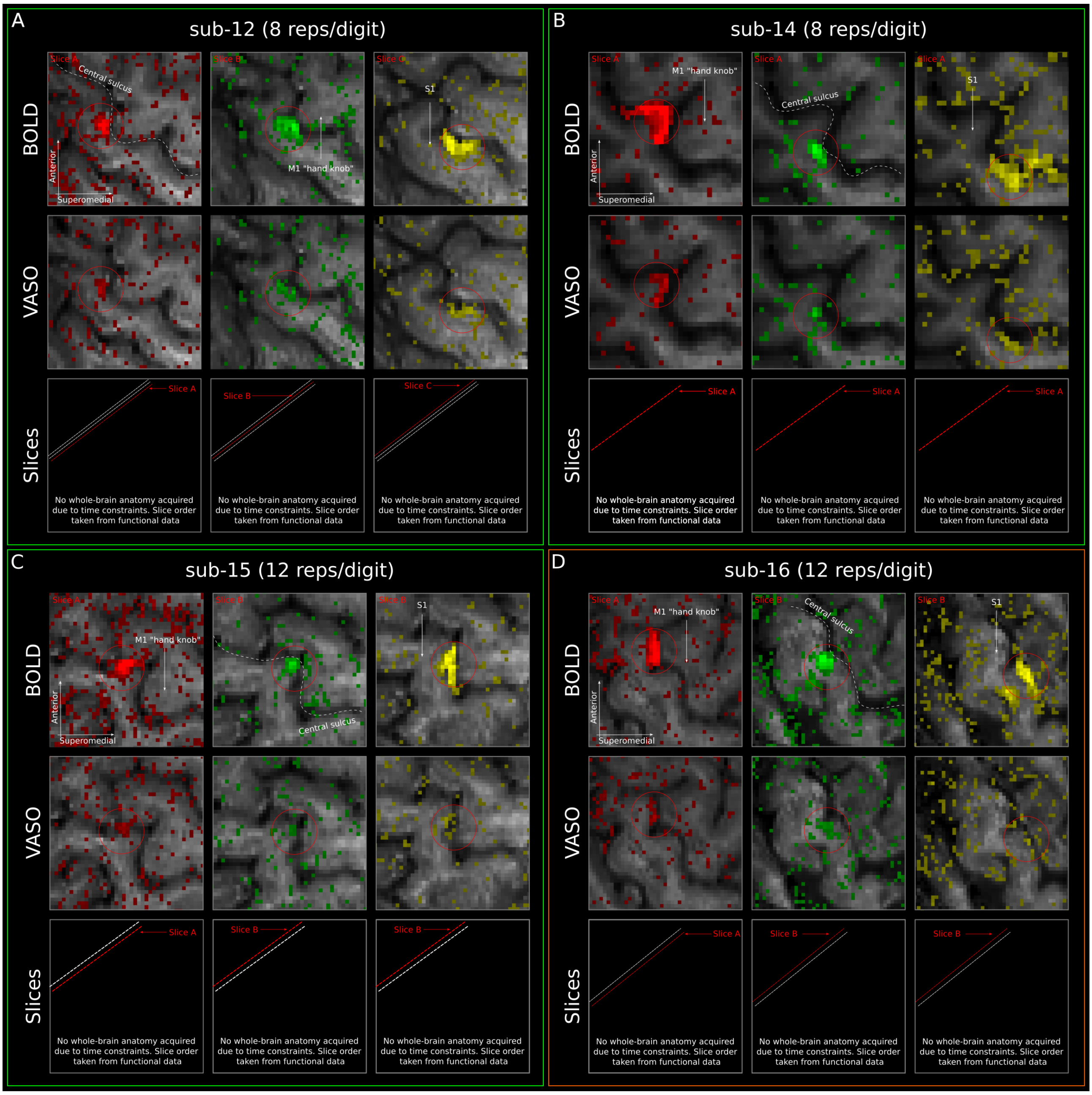

Figure S4: Stimulation results for participants sub-12-sub-16. Same as Figure 2B but for participants sub-12 (**A**), sub-14 (**B**), sub-15 (**C**), and sub-16 (**D**). A green box around the participant's plots indicates that we could identify 3/3 digit representations based on the VASO data, an orange box around the participant's plots indicates that we could identify 2/3 digit representations based on the VASO data, and a red box around the participant's plots indicates that we could identify only 1/3 digit representations based on the VASO data. Colorbars and identification of digit-clusters are the same as in Figure 2

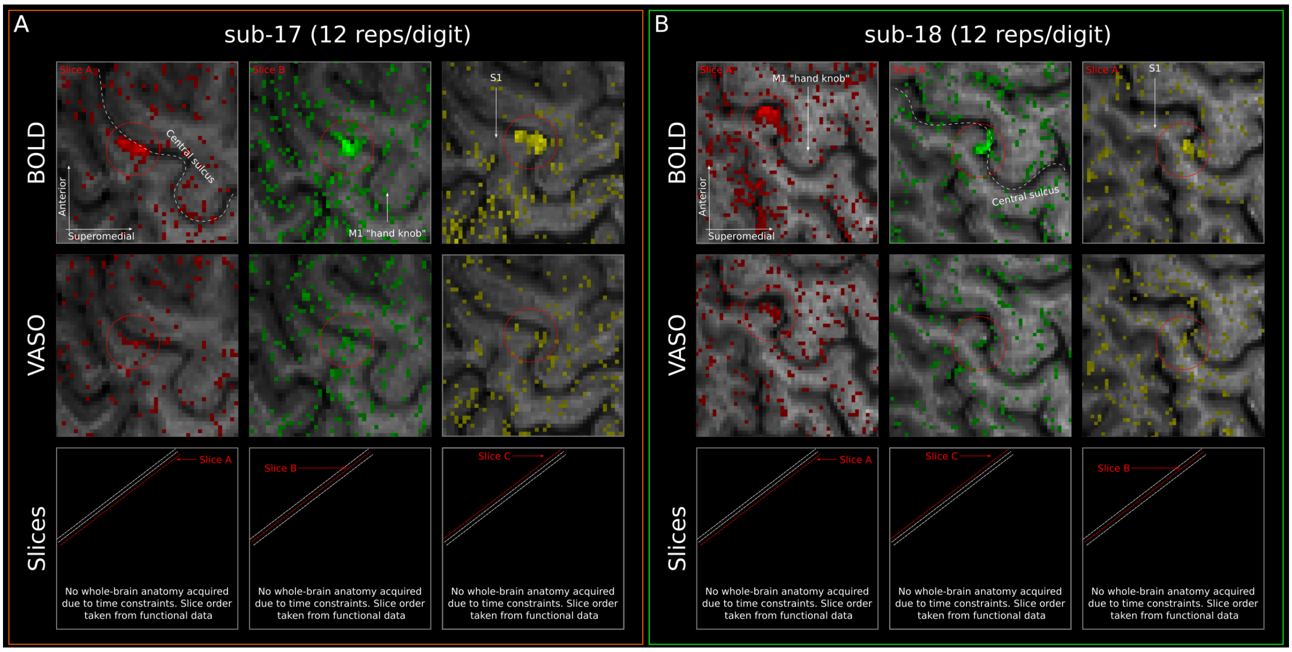

Figure S5: Stimulation results for participants sub-17 & sub-18. Same as Figure 2B but for participants sub-17 (**A**) and sub-18 (**B**). A green box around the participant's plots indicates that we could identify 3/3 digit representations based on the VASO data, an orange box around the participant's plots indicates that we could identify 2/3 digit representations based on the VASO data, and a red box around the participant's plots indicates that we could identify only 1/3 digit representations based on the VASO data. Colorbars and identification of digit-clusters are the same as in Figure 2

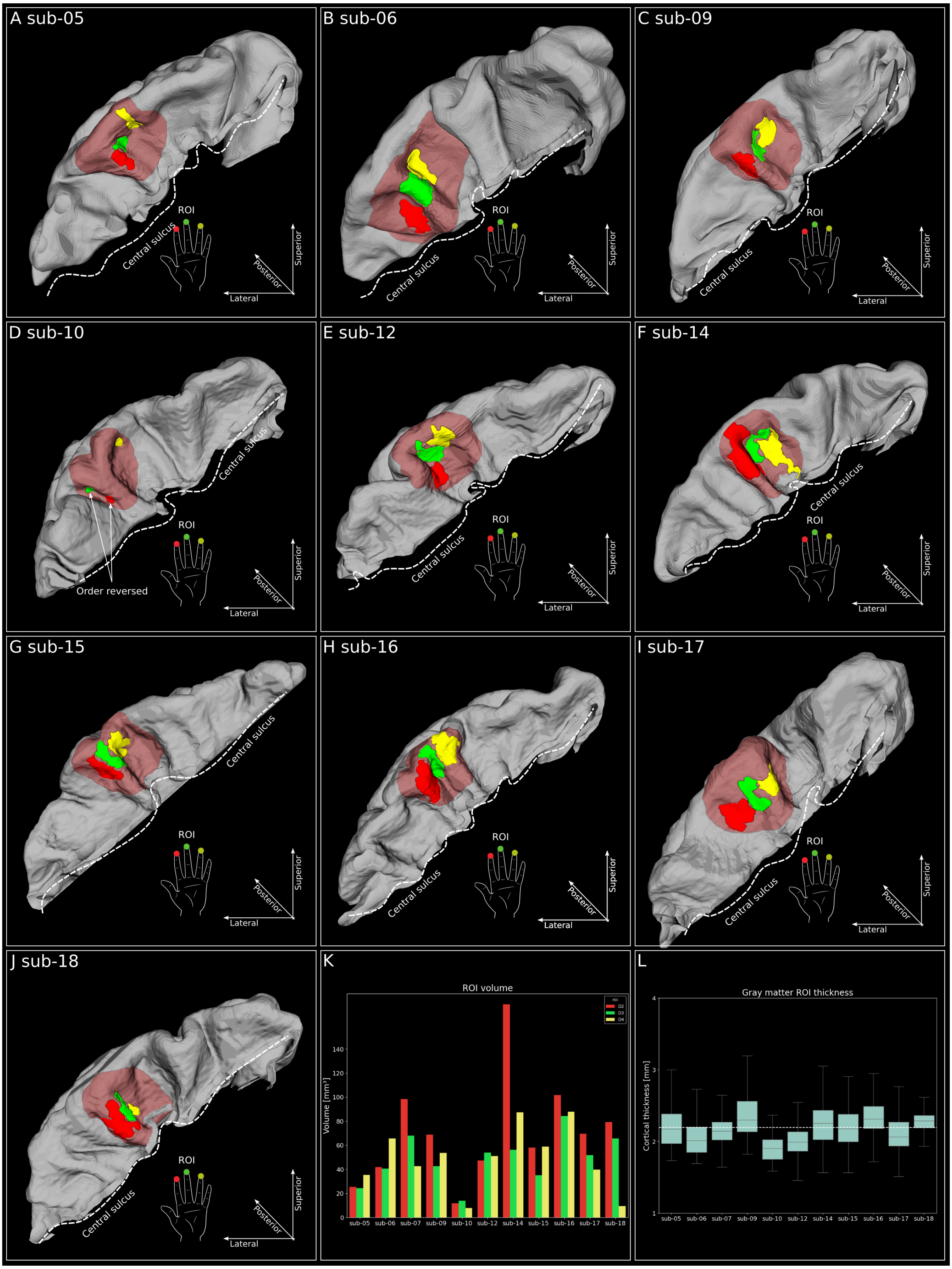

Figure S6: ROIs of individual participants. A - J) ROIs of individual participants. **K** ROI volume (mm3) of individual participants for the 3 digits separately. **L** Voxel-wise cortical thickness collapsed over ROIs for all participants independently. Dashed white line indicates mean across participants (∼2.1 mm). The box shows the quartiles, whereas whiskers show the rest of the distribution across all voxels.

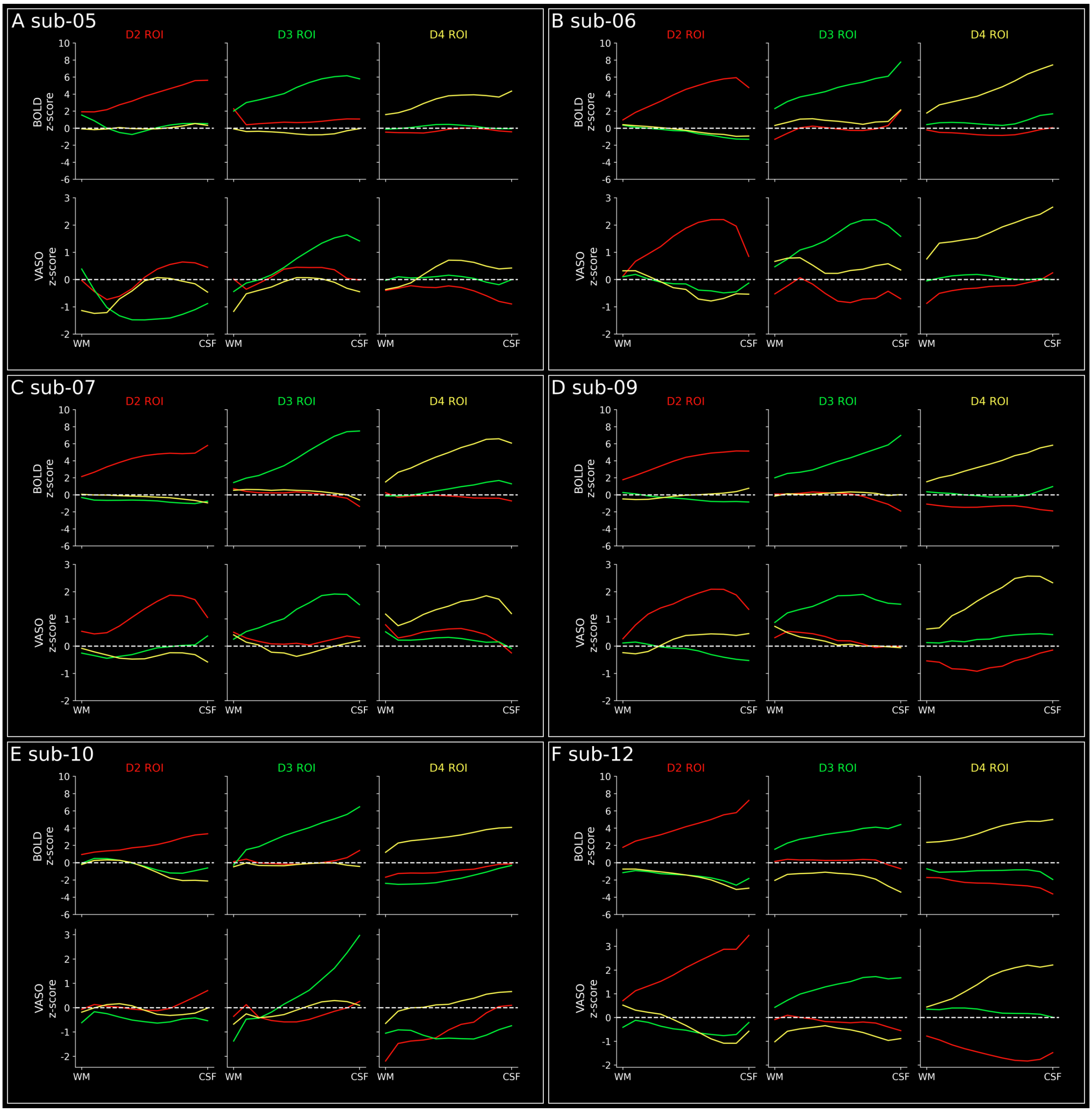

Figure S7: BOLD and VASO activation across cortical within ROIs based on BOLD for participants sub-05 - sub-12.

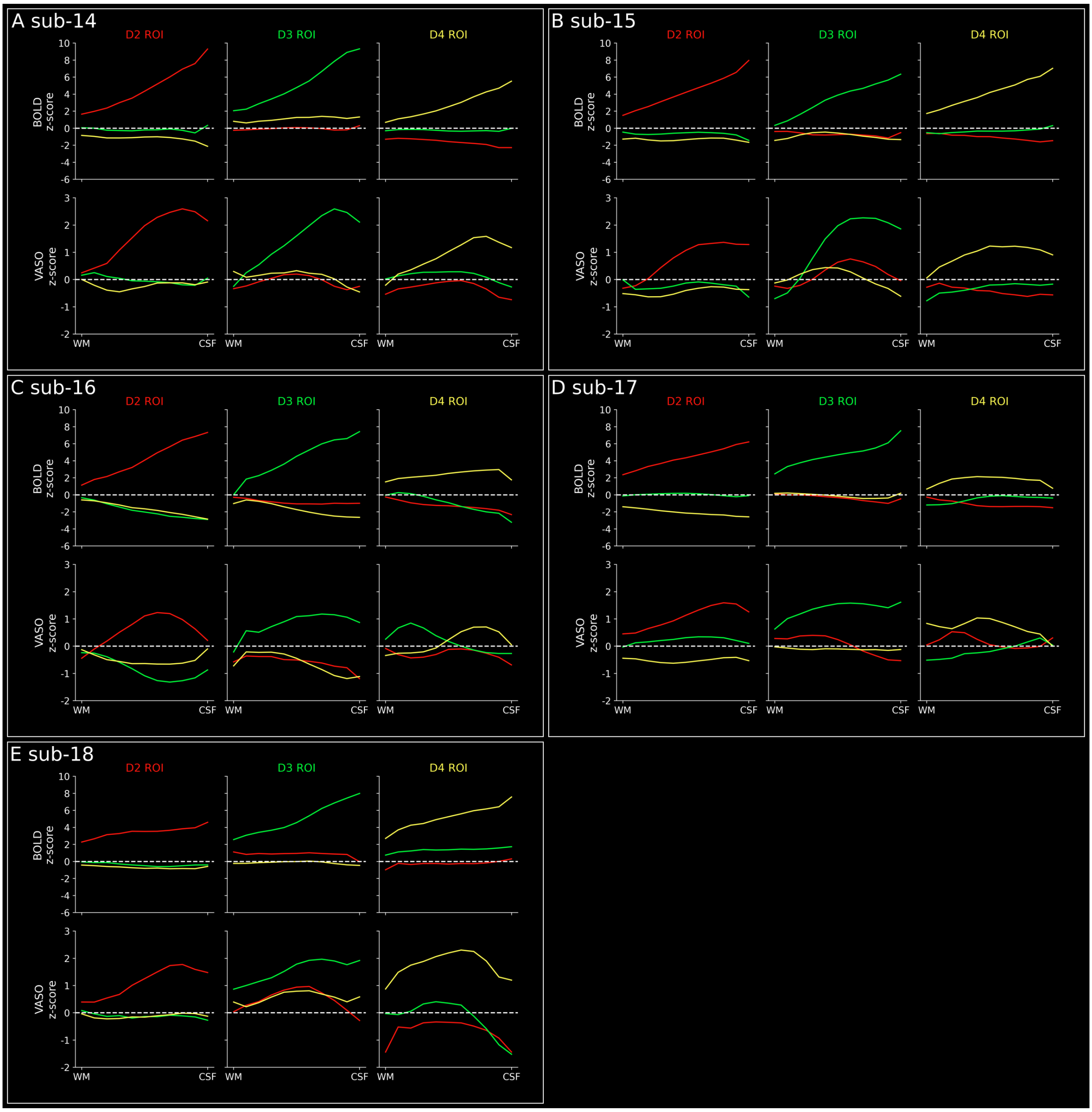

Figure S8: BOLD and VASO activation across cortical within ROIs based on BOLD for participants sub-14 - sub-18.

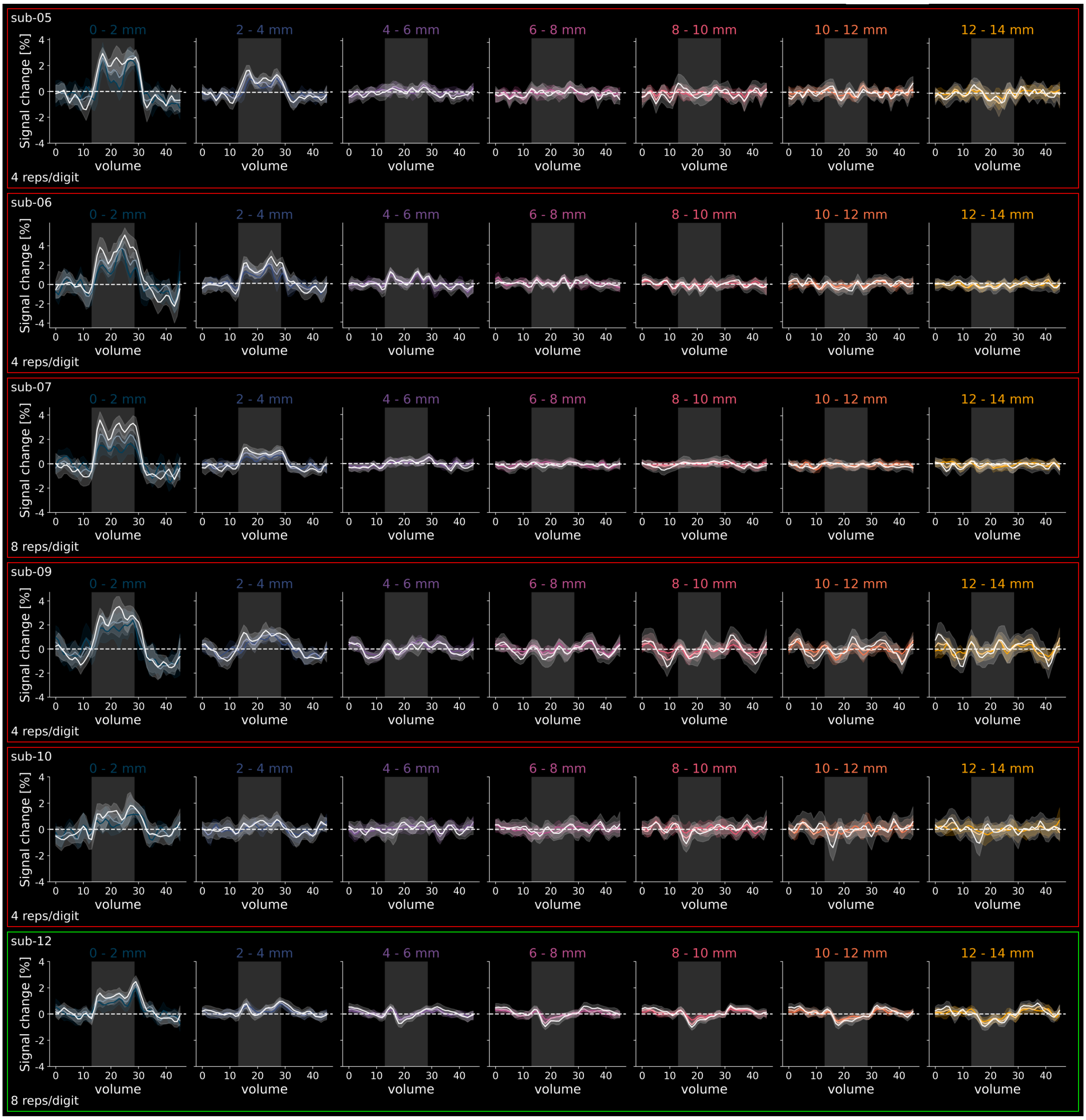

Figure S9: BOLD results of distance analysis for individual participants. Same as Figure 5B, but for participants sub-05, sub-06, sub-07, sub-09, sub-10 & sub-12 individually. A red box around a participant's plots indicates that we did not find indications of a triphasic response for this participant. A green box around a participant's plots indicates that we did find indications of a triphasic response for this participant.

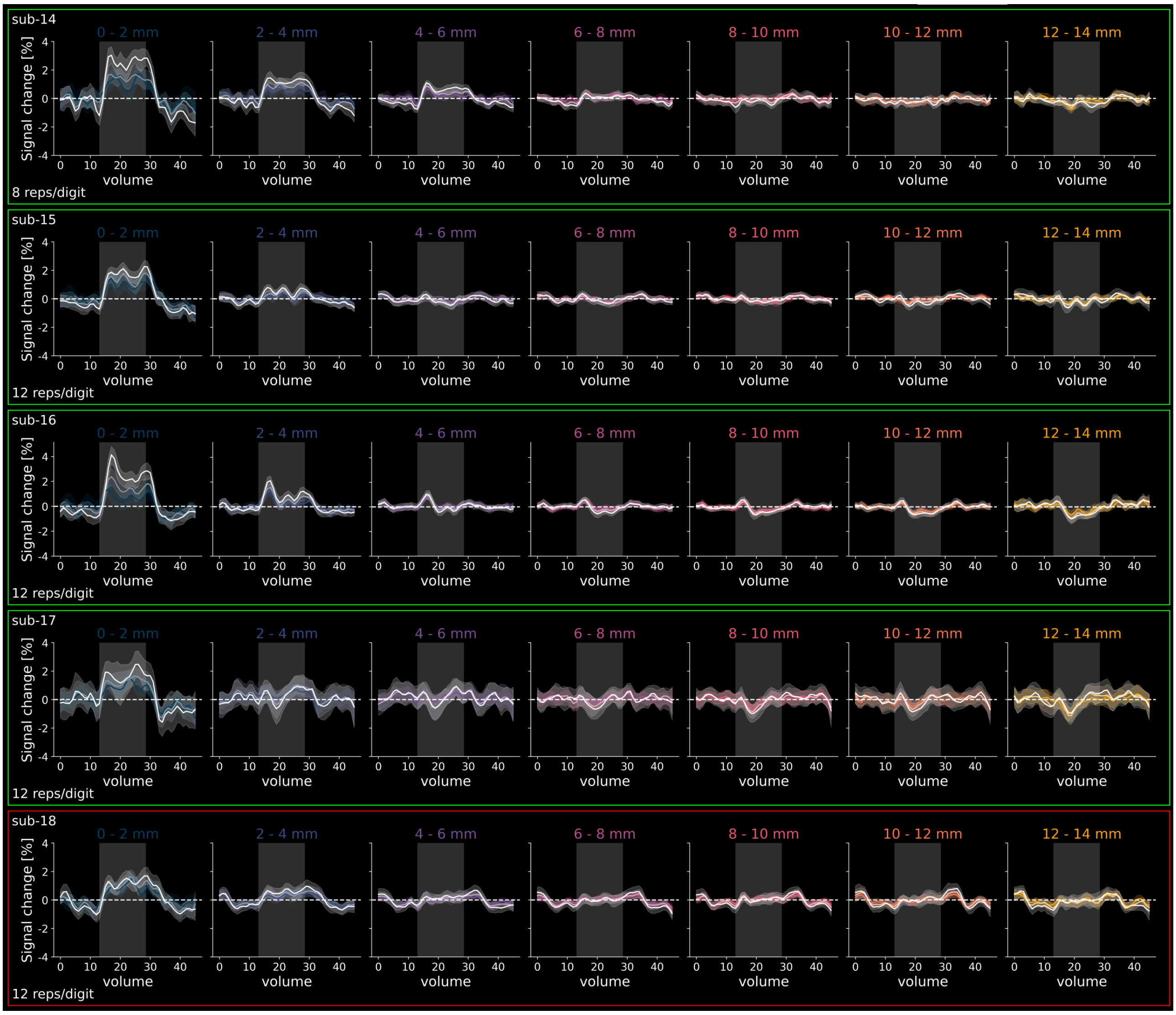

Figure S10: BOLD results of distance analysis for individual participants. Same as Figure 5B, but for participants sub-14, sub-15, sub-16, sub-17, and sub-18 individually. A red box around a participant's plots indicates that we did not find indications of a triphasic response for this participant. A green box around a participant's plots indicates that we did find indications of a triphasic response for this participant.

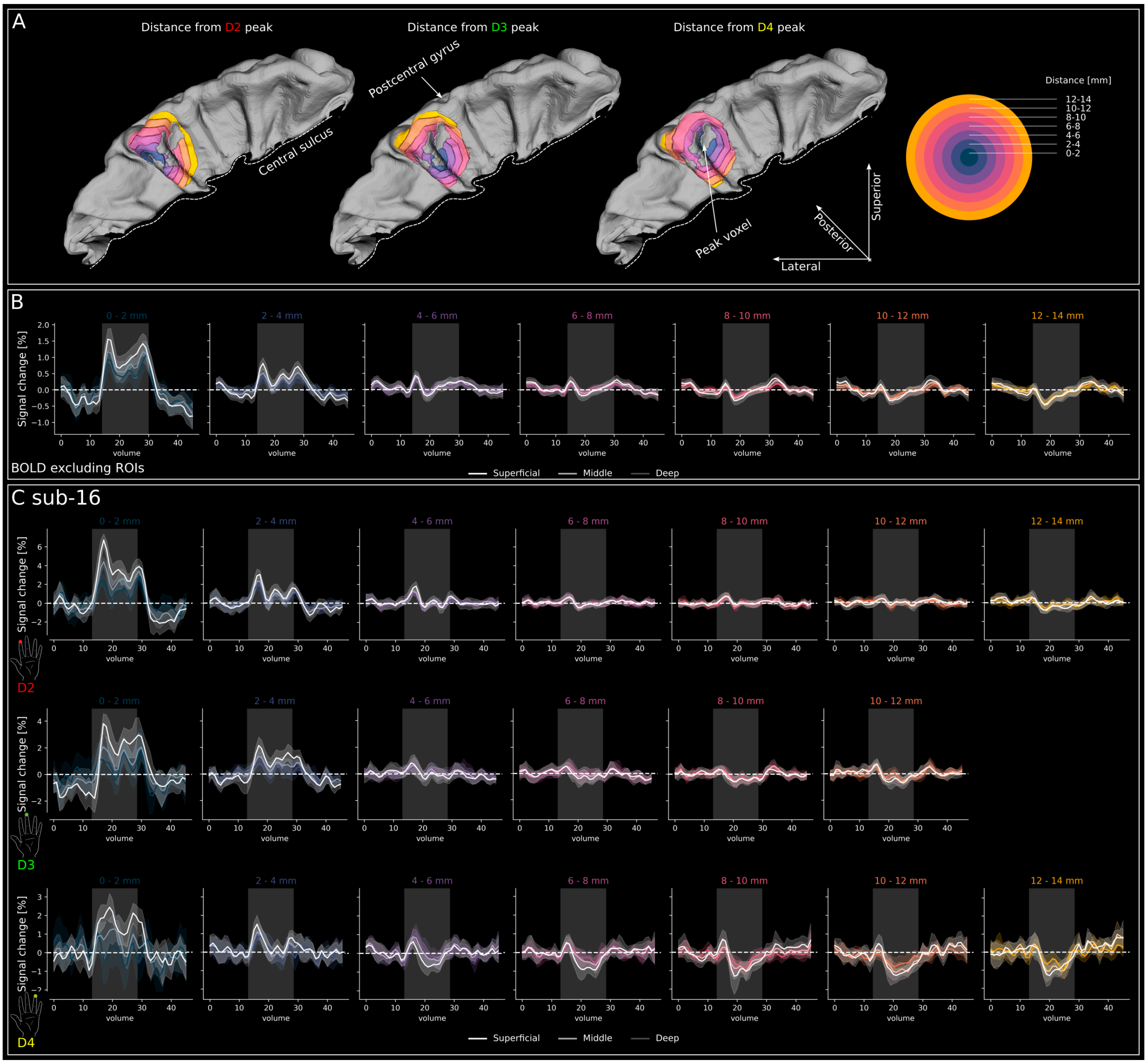

Figure S11: Additional distance-dependent analyses A Same as Figure 5A but showing the distance bins excluding the digit ROIs for participant sub-07. **B** Same as Figure 5B but excluding signal from digit ROIs. Crucially, the triphasic response is preserved. Note that the scale on the y-axis is changed with respect to Figure 5B, which makes the positive and negative deflections appear to be larger for distance bins > 2 mm. **C** Same as Figure 5A but for distance bins with respect to the 3 digits individually in one participant (sub-16). Crucially, the initial triphasic response is preserved in all individual digits. Note that the scale on the y-axis is changed between digits and, therefore, the magnitude of the positive and negative deflections appear different between rows.

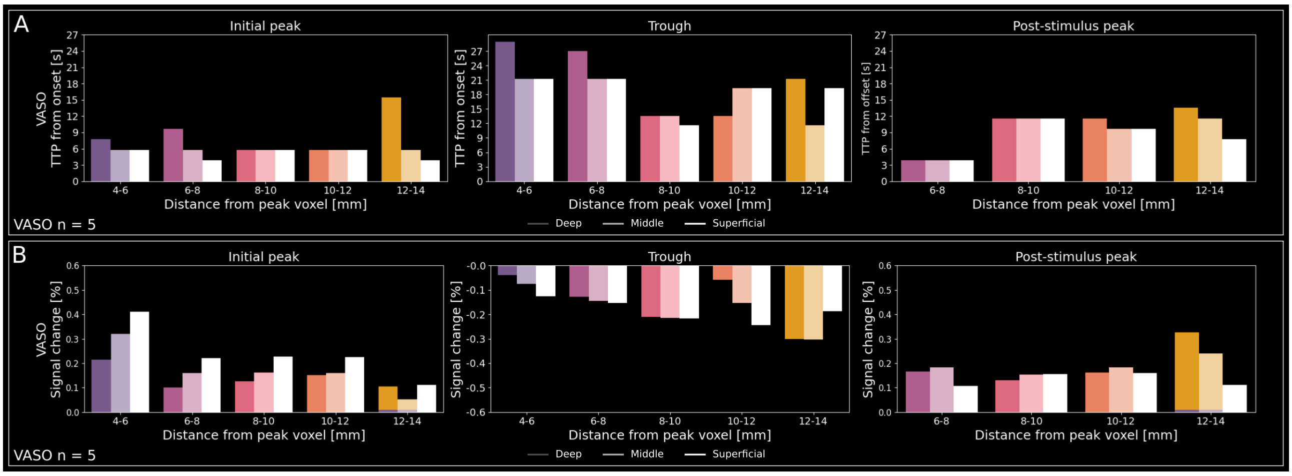

Figure S12: Quantification of layer-specific TTP and signal magnitudes for VASO across layer compartments A Same as Figure 6A but for VASO data from individual layer compartments separately. **B** Same as Figure 6B but for VASO data from individual layer compartments separately.

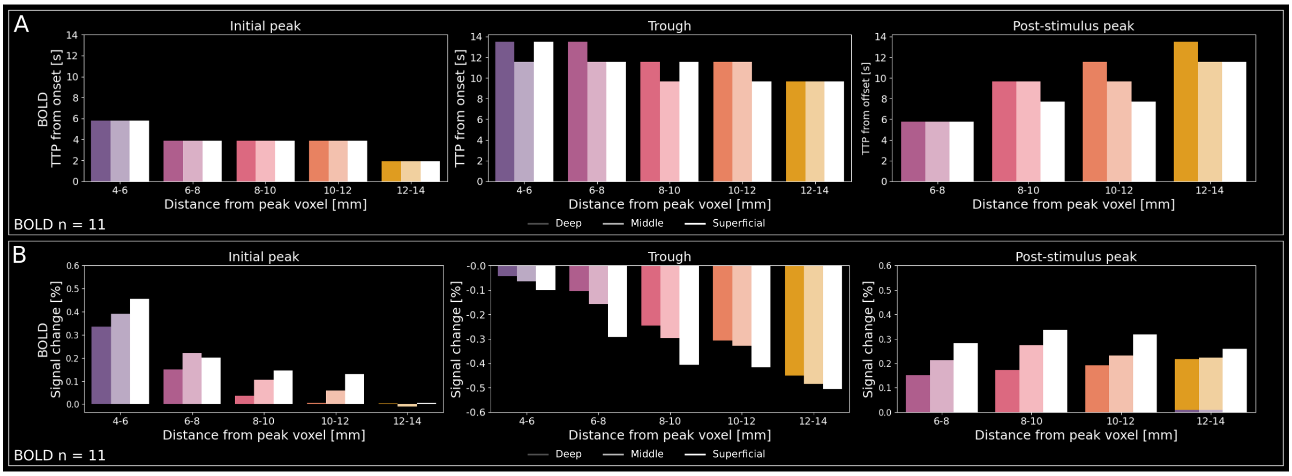

Figure S13: Quantification of layer-specific TTP and signal magnitudes for BOLD across layer compartments A Same as Figure 6A but for individual layer compartments separately. **B** Same as Figure 6B but for individual layer compartments separately.

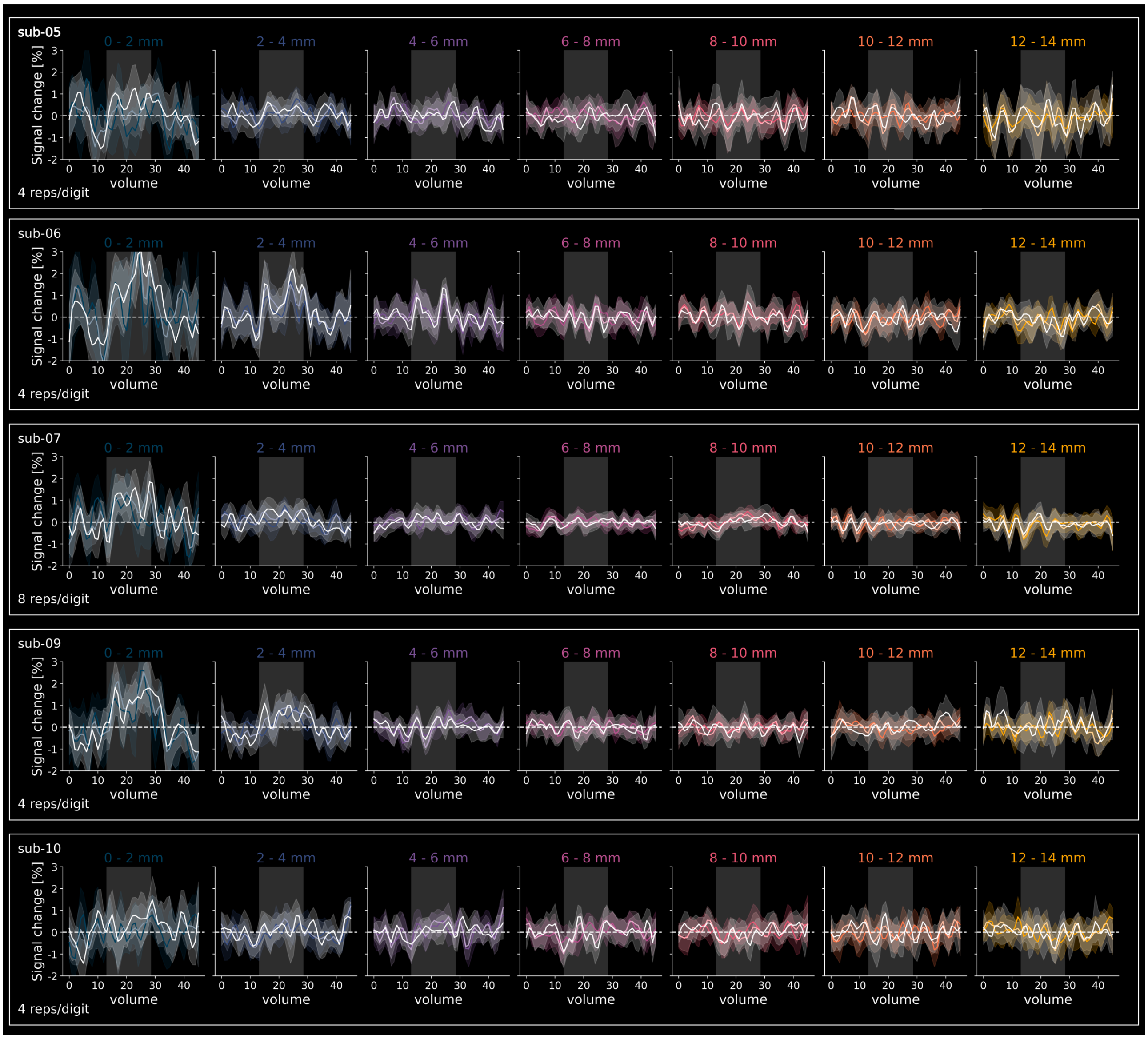

Figure S14: VASO results of distance analysis for individual participants - part 1. Same as Figure 5C, but for participants sub-05, sub-06, sub-07, sub-09, and sub-10 individually.

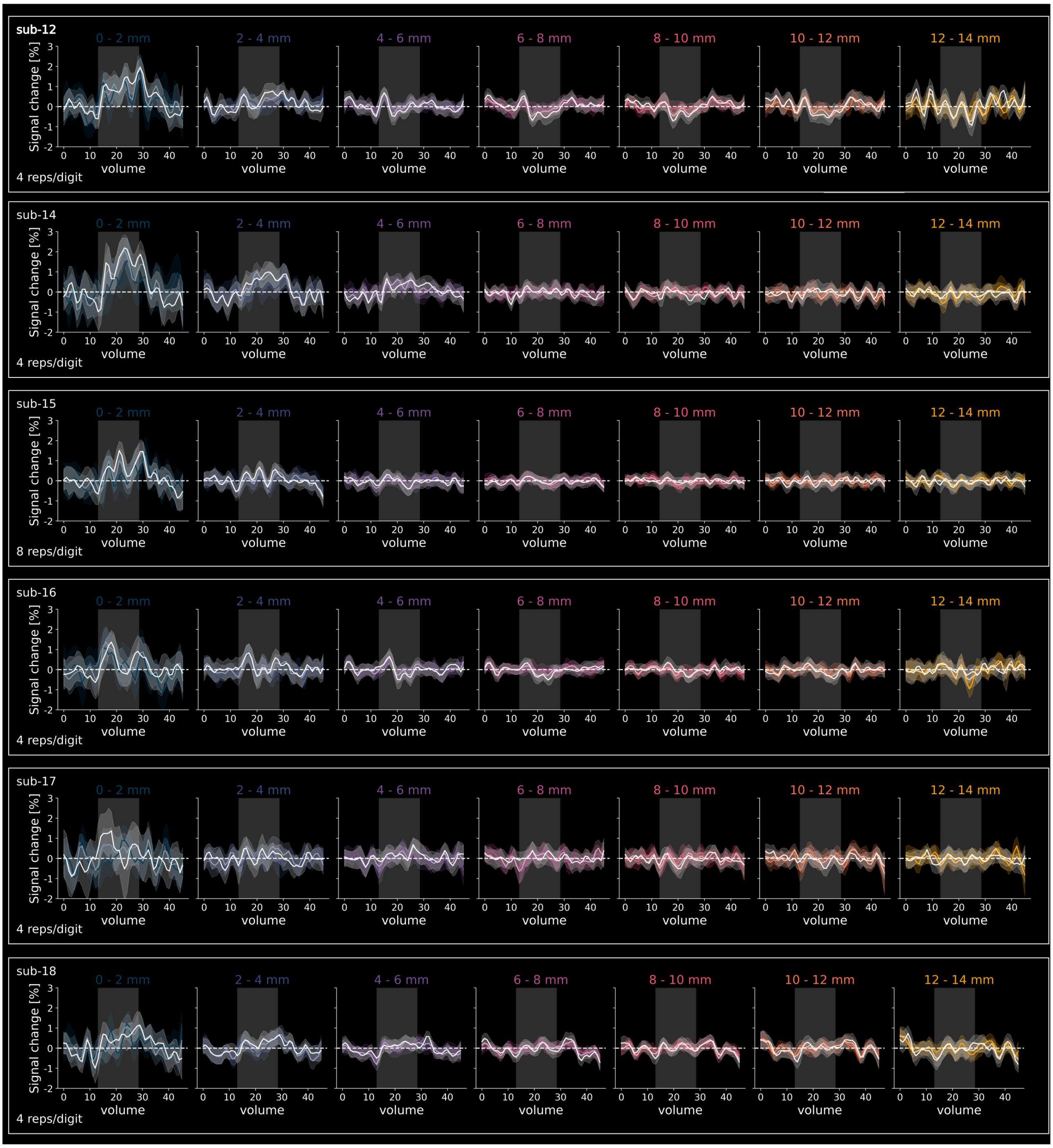

Figure S15: VASO results of distance analysis for individual participants - part 2. Same as Figure 5C, but for participants sub-12, sub-14, sub-15, sub-16, sub-17, and sub-18 individually.
